# Unwrapping the Dodecaborane Core: Structure, Electronic Properties, and Chemical Reactivity Across the Complete [B_12_I_*n*_]^−^ Series (*n* = 11–1)

**DOI:** 10.1021/jacs.6c04725

**Published:** 2026-05-21

**Authors:** Qiaoqiao Shao, Wenjin Cao, Harald Knorke, Kay Antonio Behrend, Jaskiran Kaur, Markus Rohdenburg, Daniela Volke, Hilkka I. Kenttämaa, Zhubin Hu, Zhenrong Sun, Jonas Warneke, Haitao Sun, Xue-Bin Wang

**Affiliations:** † State Key Laboratory of Precision Spectroscopy, School of Physics, 12655 East China Normal University, Shanghai 200241, China; ‡ Physical Sciences Division, 6865 Pacific Northwest National Laboratory, 902 Battelle Boulevard, P.O. Box 999, Richland, Washington 99352, United States; § Wilhelm-Ostwald-Institut für Physikalische und Theoretische Chemie, 9180 Universität Leipzig, Linnéstr. 2, 04103 Leipzig, Germany; ∥ James Tarpo Jr. and Margaret Tarpo Department of Chemistry, 311308 Purdue University, 560 Oval Drive, West Lafayette, Indiana 47907, United States; ⊥ Institute of Bioanalytical Chemistry, Faculty of Chemistry and Center for Biotechnology and Biomedicine, Universität Leipzig, 04103 Leipzig, Germany; # Leibniz-Institut für Oberflächenmodifizierung e.V. (IOM), 04318 Leipzig, Germany; ∇ Collaborative Innovation Center of Extreme Optics, Shanxi University, Taiyuan, Shanxi 030006, China

## Abstract

Successively stripping the exohedral substituents from the stable *closo*-dodecaborate anion [B_12_I_12_]^2–^ results in structural transformations of the icosahedral B_12_ into a (quasi-)planar unsubstituted B_12_ unit. Previous studies have revealed that [B_12_I_11_]^−^ to [B_12_I_8_]^−^ ions contain a closed B_12_ unit, while [B_12_I_7_]^−^ is the first ion in the series with an opened B_12_ unit. Further transitions in geometry, electronic structure, and chemical reactivity across the whole range of fragments [B_12_I_*n*_]^−^ (*n* = 11–1) remain elusive. Herein, we report a systematic investigation to explore the chemical properties of these intermediate structures by using negative ion photoelectron spectroscopy (NIPES), ion mobility spectrometry (IMS), gas-phase ion–molecule reactions, and quantum chemical calculations. [B_12_I_*n*_]^−^ ions can be categorized into three groups: (1) very reactive ions with intact (quasi-)­icosahedral B_12_ cages (*n* = 11–8), (2) less reactive ions with open structures (*n* = 7–4), and (3) almost unreactive ions with (quasi-)­planar structures (*n* = 3–1). Preparative mass spectrometry shows that ions with the unsaturated B_12_ core (category 2) tend to form smaller, fully substituted *closo*-borate anions [B_*m*_X_*m*_]^2–^ (*m* = 6–11 with X = I, H, OH) on surfaces. In contrast, ions in category 3 cannot be found on the surface and apparently decay into volatile products. This research provides fundamental insights into the physical and chemical properties of B_12_ units depending on their substitution level and paves the way for the rational design of boron-rich compounds by using unconventional [B_12_I_*n*_]^−^ building blocks.

## Graphical abstract

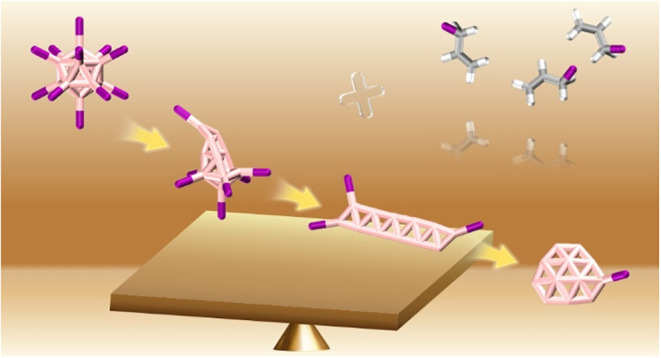

## Introduction

Boron-based molecules, clusters, and ions have received considerable attention due to their special bonding properties and diverse geometric structures, including one-dimensional boron atomic chains, two-dimensional boron sheets, and three-dimensional (3D) boron nanoribbons, nanotubes and core–shell fullerenes.^1–4^ Polyhedral boranes constitute the most common textbook examples of boron compounds with 3D structures. Closed boron polyhedra with each boron atom bound to a substituent X exist in dianionic form (*closo*-borate dianion). The most widely studied example is the *closo*-dodecaborate dianion [B_12_X_12_]^2–^ where X can be, for example, H, halogen, OR (R = organic residue), or CN groups.^5–7^ These dianions exhibit exceptionally high structural and electronic stability resulting from their icosahedral symmetry, 3D aromaticity and delocalized charge. The choice of X enables the physical and chemical properties of *closo*-dodecaborates to be tuned, e.g., oxidation potentials:^8^ [B_12_(CN)_12_]^2–^ is the most electronically stable dianion to date,^7^ while OR-persubstituted clusters can be readily oxidized and isolated as radical anions or even neutral compounds.^9^ Compounds containing dodecaborate dianions have been researched for various applications, such as boron neutron capture therapy for cancer treatment,^10,11^ transmembrane drug carriers based on superchaotropic properties,^12,13^ highly selective chemical separations,^14^ activation fuel,^15^ electrochemical ion batteries,^16^ and stabilization of reactive cations for catalysis and chemical synthesis.^17–19^

Unlike the prevalent 3D motifs in bulk boron, pure anionic boron clusters (B_*n*_^–^) with *n* ≤ 24 exhibit 2D planar or quasi-planar structures.^20–23^ This unique structural characteristic can be explained by σ- and π-aromaticity or antiaromaticity within these elemental clusters. Particularly B_12_^–^ features a bowl-shaped quasi-planar structure, in stark contrast to the well-known icosahedral B_12_ cage found in *closo*-borate dianions.^23^ Neutral B_12_ has a large HOMO–LUMO gap of ∼2 eV,^24^ even larger than that of C_60_,^25^ implying low chemical reactivity. Though both theoretical and experimental studies have been carried out on the structures of unsubstituted boron clusters,^26,27^ only limited knowledge about their chemical reactivities (published^28,29^ three decades ago) was available in the literature until recently. However, the topic regained research interest very recently when reactions of B_12_^+^ and B_13_^+^ with CO and H_2_O were studied.^30^

Collision-induced dissociation (CID) of [B_12_I_12_]^2–^ enables successive cleavage of iodine substituents (cleavage of one iodide anion followed by multiple iodine radicals).^31^ In the final step, [B_12_I_1_]^−^ dissociates into I^–^ and neutral B_12_.^31,32^ The icosahedral [B_12_X_12_]^2–^ ions, as well as the quasi-planar unsubstituted neutral B_12_ compound, represent two stable end points of a series of possible [B_12_X_*n*_]^−^ structures. Several experimental and theoretical studies aimed at identifying the level of substitution that led to the opening of the B_12_ scaffold into a nonicosahedral geometry upon sequential removal of substituents.^32–34^ After the mass spectrometric detection of the [B_12_I_*n*_]^−^ series in 2011,^31^ a theoretical investigation in 2012 predicted that the B_12_ cage structure at *n* = 6–4 should be open.^32^ In contrast, gas-phase infrared spectroscopy in combination with computational investigations revealed in 2015 that open structures must be present at *n* = 7.^33^ This is further supported by an ion deposition study in 2021, showing that [B_12_I_7_]^−^ reacts differently at interfaces than the more substituted ions *n* = 8–11.^35^ The chemical properties of fragment ions with icosahedral B_12_ units (*n* = 11–8) have been examined intensively by using gas-phase ion chemistry and preparative mass spectrometry.^7,31,35–41^ In contrast, less data exist on the chemical properties of [B_12_I_*n*_]^−^ ions with *n* = 7–1 exhibiting open B_12_ units. Early computational work reported optimized geometries for these ions but the reported minimum structures were later shown not to be generally predictive for the real (experimental) structures.^32,33^ Additionally, properties such as electronic structures and chemical reactivity remain unknown. Despite extensive preliminary work, a fundamental understanding of the structure–reactivity relationship of [B_12_I_*n*_]^−^ ions beyond *n* = 8 (open B_12_ cage) is lacking. A detailed understanding of the structural, electronic and reactivity features of [B_12_I_*n*_]^−^ ions dependent on their substitution level *n* would (i) provide new insights into physical and chemical diversity of the most common boron unit—the B_12_ unit, and (ii) lay the basis for the rational design of new compounds for application in medicine and material science^10,42^ using unconventional reaction intermediates.

Therefore, we explore the electronic structure and reactivity of the entire [B_12_I_*n*_]^−^ (*n* = 11–1) ion series by using gas-phase negative ion photoelectron spectroscopy (NIPES), ion mobility spectrometry (IMS), gas-phase ion–molecule reactions, ion soft-landing experiments and computational investigations. Our results demonstrate that the ion structures ranging from icosahedral B_12_ to planar B_12_ units can be categorized into three groups, intact icosahedral cages (*n* = 11–8), open structures (*n* = 7–4), and planar or quasi-planar structures (*n* = 3–1) with distinct electronic and chemical features. NIPES shows that the electronic structures of the fragment anions alternate between closed-shell ions (odd *n*, even electrons) and open-shell radicals (even *n*, odd electrons). Both the electronic structure and geometric topology profoundly influence their reaction behaviors both in the gas phase and in the condensed phase.

## Results and Discussion

CID of [B_12_I_12_]^2–^ ions results in a distribution of [B_12_I_*n*_]^−^ ions dependent on the applied collision energy as shown in Figure S1. In general, fragments with even numbers of iodine atoms (*n* = 2, 4, 6, 8, and 10) are radical anions and observed in lower abundance in the mass spectra than their neighbors with odd numbers of iodine atoms (*n* = 1, 3, 5, 7, 9, and 11). The 20 K NIPE spectra of [B_12_I_*n*_]^−^ (*n* = 11–1) measured with 157 nm photons are shown in Figure 1a and indicate a trend of overall decreasing electron binding energy (eBE) with a decreasing number of iodine substituents. The ground state vertical detachment energy (VDE), measured from the peak position of the first lowest eBE band in each spectrum, is marked with a gray-dotted line and also shows an odd–even pattern, with radical ions exhibiting smaller VDE (less electronically stable, see Table S1 and Figure S2). The intensity trend in the CID mass spectra in Figure S1 and the VDE trend in NIPES spectra in Figure 1a demonstrate that the radical ions are less stable than their even-electron neighbors in the series, with respect to both electron and iodine loss. The [B_12_I_7_]^−^ NIPE spectrum has a distinct pattern different from its predecessors with a doublet peak resolved in eBE = 4.7 to 5.5 eV and a plethora of peaks congested in the high eBE region, which deviates from the band topology consistently observed for *n* = 11–8. The new spectral pattern (red shaded in Figure 1a) largely remains for *n* = 6, 5, and 4. Starting from *n* = 3, the general band structure again changes significantly, becoming less congested and exhibiting better-resolved bands. This trend in photoelectron spectra suggests that [B_12_I_*n*_]^−^ fragments may be divided into three categories: *n* = 11–8 (blue), *n* = 7–4 (red), *n* = 3–1 (yellow). Such a categorization is further supported by the structural evolution within the series, as shown by ion mobility spectrometry (IMS) investigations (Figure 1b). A rather monotonous decrease in drift time was observed for *n* = 11 to 8, which can be associated with stepwise loss of iodine upon CID without substantial structural changes to the ions’ backbone. Note that multiple additions of water and nitrogen background gas molecules to the vacant sites of the boron icosahedral are observed. Note that [B_12_I_11_]^−^ is highly reactive and predominantly its N_2_ adduct could be observed in these experiments. Ions between *m*/*z* 1020 and 1100 contain only seven iodine atoms with a various number of background gas molecules but are formed by iodine loss from adduct ions of [B_12_I_8_]^−^ (see Table S2). The trend is interrupted at *n* = 7 and an increase in drift time is observed for [B_12_I_7_]^−^. Ion signals at the same *m*/*z* but with different drift time values show the coexistence of structurally well distinguishable isomers. Inserts in Figure 1b display the two-dimensional ion-mobility traces for the ions with *n* = 7, 6, and 3 evidencing the coexistence of such isomers. From *n* = 7 onward, the overall decreasing trend in drift time shows some deviations from a linear trend (broader red shaded area). This may indicate that in addition to iodine loss, some structural transformations of the boron core occur. The drift time does not decrease significantly upon loss of a single iodine atom from [B_12_I_4_]^−^. However, with further iodine cleavage (from *n* = 3 to *n* = 1), the rate at which the drift time decreases becomes steeper. Therefore, both NIPES and IMS of the whole series suggest three categories of fragments, which show characteristic geometric features and electronic structures.

**Figure 1:**
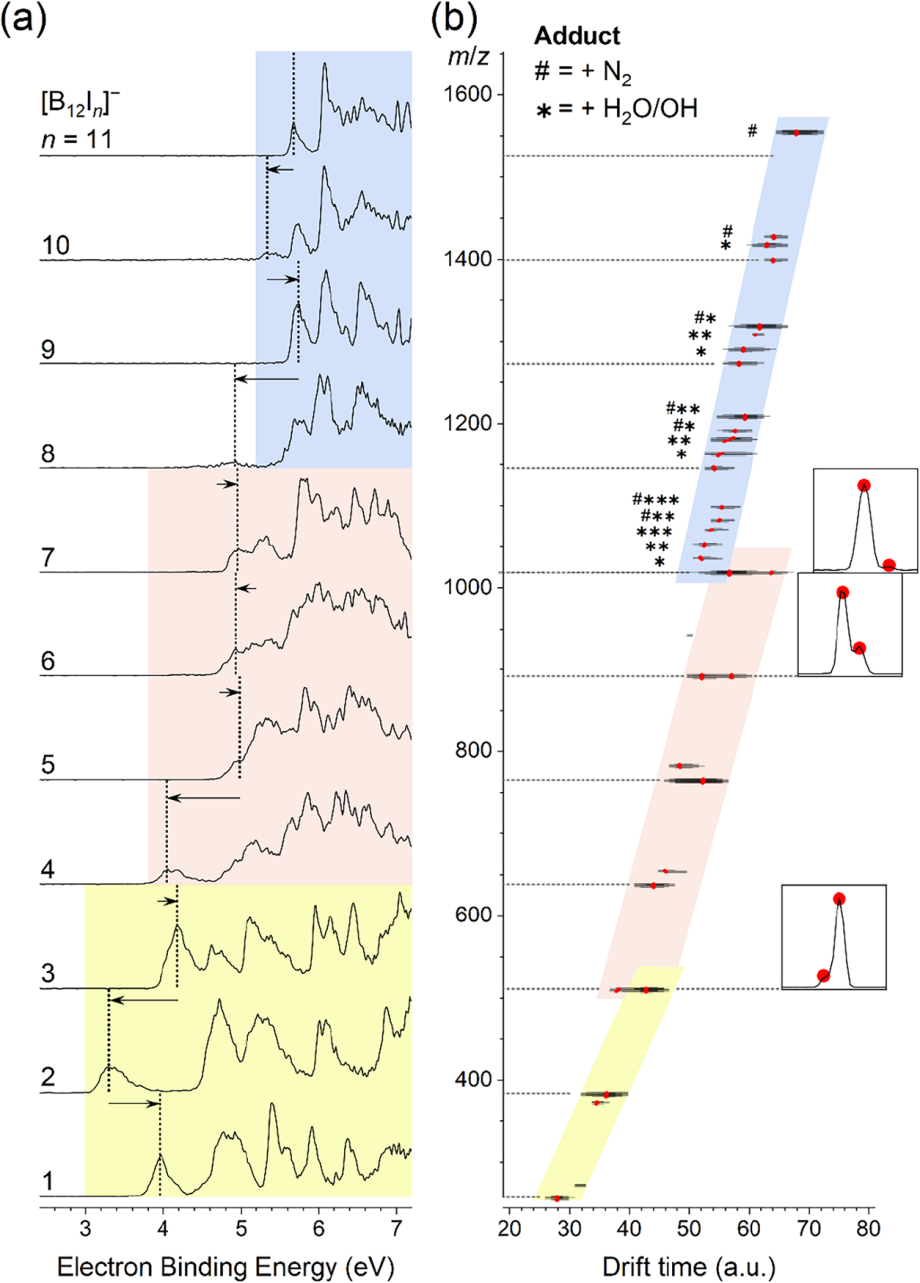
(a) The cryogenic 20 K 157 nm NIPE spectra of [B_12_I_*n*_]^−^ (*n* = 11–1). Vertical detachment energies (VDEs) are marked with a dotted line and black arrows indicate shift in VDE with respect to neighboring substitution levels. (b) Ion mobility mass spectrometry results showing the development in drift time dependent on *n*. 200 bins make up each mobility experiment. Ions originating from the binding of background gases nitrogen and water are marked with # and *, respectively. Inserts show the separation of isomers in a 2D-depiction (drift time vs abundance). A list of detected *m*/*z* values and molecular formula assignments can be found in the Supporting Information (SI), Table S2.

To assign NIPE spectra to ion structures and to correlate IMS results with the topology transformation of the B_12_ core from an icosahedron (at high substitution level) to a plane geometry (without substituents), [B_12_I_*n*_]^−^ (*n* = 11–1) anions were explored computationally via “top-down” stepwise “iodine atom peeling” and “bottom-up” atomic recombination (Figure S3). We note that the pioneering computational investigations in 2012 ^32^ could not find all low-lying isomers. Further, the computational level of previously published results^32^ does not allow a comparison with NIPES data nor IMS results (details are provided in the SI). Figure S4 shows a comprehensive set of the low-lying isomers computed herein. Fully enclosed cage-like structures (category 1) are labeled “C*-x*_(*n*)_”, opened structures are labeled “O*-y*_(*n*)_” (category 2), and planar ones are labeled “P*-z*_(*n*)_” (category 3), respectively. The variables *x, y*, and *z* label the isomers in their energetic order within the respective category and the index (*n*) represents the number of iodine substituents. To compare the trend in the IMS results with the computationally predicted structures, collision cross-section (CCS) values were calculated from the experimental drift times by using a polyalanine calibration standard. Since precise CCS value determination with the IMS method employed here is dependent on how comparable in nature are the ions used for calibration and the analyte ions (no ions with known CCS values similar to [B_12_I_*n*_]^−^ are available to us), the obtained CCS values listed in the SI, Table S2 do not represent reliable absolute values and are only used for the purpose of comparing trends with computed CCS values of the structures. The computed CCS values were linearly scaled for the comparison shown in Figure 2 (middle). Details of the procedure are described in Table S3.

**Figure 2:**
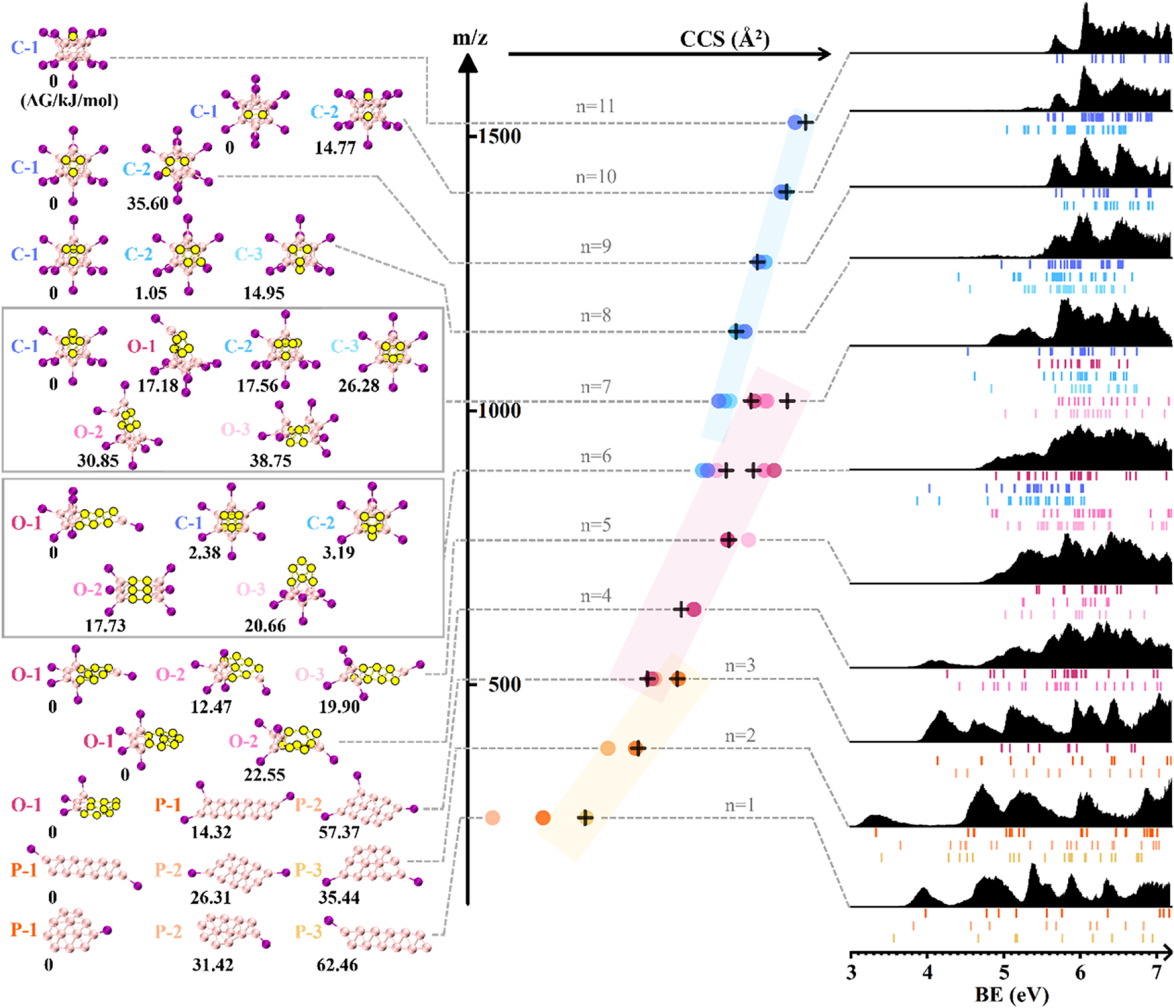
Lowest-lying isomers of [B_12_I_*n*_]^−^ (*n* = 11–1) (left), their computed CCS (solid circles) (middle) and DOS stick spectra (right) compared with the experimental CCS (black plus) and experimental NIPE spectra (black shaded). All vacant boron sites in the cage (category 1) and open structures (category 2) are highlighted in yellow. All isomer labels, CCS circles and DOS sticks are color matched.

Selected energetically favorable structures are shown in Figure 2 (left). A color code is used to compare the match of computed CCS (Figure 2, middle) and orbital density of states (DOS, Figure 2, right) with the experimental data introduced in Figure 1. Note that the geometry of the icosahedral structures (category 1) can be considered well evidenced as the calculated CCS values are similar for different isomers, justifying a scaling factor for computed CCS values that aligns experimental and computational values for these structures (blue shaded region). For *n* = 11 to 8, the B_12_ cage structure remains intact. Iodine loss occurs at adjacent positions for *n* = 11 to 9. [B_12_I_9_]^−^ features an iodine-deficient boron atom triangle. Further cleavage of the B–I bond at the opposite side of the cluster leads to the most stable [B_12_I_8_]^−^ isomer. While the absolute CCS values for *n* = 11–8 cannot be used to determine which isomers are present, the comparison of computed DOS with NIPES leads to the conclusion that the C-1 structures are dominant. Note that in contrast to a nearly perfect agreement between the calculated and experimental VDE values for the *n* = 11 and 9 anions, both closed-shell species, a deviation of roughly 0.2 eV is observed between the DLPNO–CCSD­(T)-computed VDE and the experiment for isomer C-1 of *n* = 10, which is most likely due to the larger deviation inherent in computing open shell system as previously reported.^43^ At *n* = 7, low-lying open structures were found. Our investigation revealed an energetically lowest-lying open structure isomer O-1_(7)_, which was not discovered by previous computational investigations.^33^ However, the lowest-lying cage structure (C-1_(7)_) is still preferred at *T* = 20 K. It is therefore surprising that both IMS and NIPES do not indicate the presence of the closed cage isomer C-1_(7)_ in the isomer mixture that contains open structures. C-1_(7)_ would have a ca. 0.5 eV smaller VDE than the measured value and the whole band from eBE = 4.8 to 5.4 eV cannot be explained by this isomer (see its predicted DOS in Figure 2, right (dark blue)). It should be noted that low-lying open structures (O-1_(7)_ to O-3_(7)_) are not distinguishable with our IMS instrumental resolution (compare computed values with the width of experimental signal in the inset in Figure 1) but the comparison of DOS and NIPES spectra indicates that the O-3_(7)_ isomer is present in the mixture since its DOS aligns well with the measured VDE and first spectral bands. For [B_12_I_6_]^−^, an open structure constitutes the global minimum. While closed B_12_ structures (category 1) are low in energy also, they were not observed experimentally. The CCS of the main isomer is apparently best assigned by O-3_(6)_, which forms as the lowest energy structure when iodine is removed from O-1_(7)_ (Figure S5). The computed CCS of O-2_(6)_ can be roughly matched with the second signal at higher CCS, and may be formed by iodine loss from O-3_(7)_. Although our IMS measurements cannot separate isomers for *n* = 5 and 4, the width of the signal indicates that several isomers with similar CCS are present (Figure S6). Although the boron icosahedron structure is open from *n* = 7 onward, the structures down to *n* = 4 still contain spherical structures at the iodinated parts and some structures show extruded flat boron sheets, see, for example, O-1 for *n* = 6–4 (Figure 2, left). At *n* = 3, a planar structure (P-1) becomes energetically similar to the global minimum (O-1) and constitutes the dominant component in the experimentally obtained isomer mixture. Upon further removal of iodine, P-1_(2)_ and P-1_(1)_ constitute the global minima. The comparison of DOS with NIPES spectra indicates that they are the prevailing isomers obtained experimentally. We note that in order to maintain the correlation between experimental and calculated CCS values, we needed to apply a different linear scaling for the computed CCS values for *n* = 3–1. This may be justified by the change in the geometric and chemical properties of the ions upon transfer to planar structures, affecting calibration. Aligning the calculated CCS value of planar minimum P-1_(3)_, for which the calculated DOS also fits the major features of the experimental NIPES data well, with the most abundant IMS signal, confirms that the major IMS signal for *n* = 2 can be rationalized with the calculated CCS value of the global minimum P-1_(2)_. For *n* = 1, a clear structural assignment is difficult due to deviations in the trend of calculated and measured IMS data, but the global minimum structure (P-1_(1)_) shows best agreement with respect to measured NIPES data and calculated DOS.

Although the computed lowest energy structures are in accordance with the experimental results for ions in categories 1 and 3, a mixture of isomers is present in the ions in category 2 and enthalpic arguments alone are not sufficient to predict the relative abundances of the different isomers. This finding for category 2 ions rationalizes why previous computational investigations without spectroscopic data^32^ were unable to reliably predict the substitution level (number of iodine substituents) that result in opening of the B_12_ core (transition from category 1 to category 2 ions). Instead, our investigation does not only confirm that the first open structures are present at *n* = 7 as concluded based on previous IRPD investigations^33^ but also reliably shows that closed structure isomers of [B_12_I_7_]^−^ do not play a significant role in the formed isomer mixture, despite their lower enthalpy at 20 K. This counterintuitive result may be rationalized based on thermal effect, given the fact that CID fragments are formed by vibrational excitation (corresponding to high temperatures reported to be >1000 K).^44^ As shown in Figure 3, at different temperatures, the relative Δ*G*s of the cage-like structures remain similar, while those of open structures decrease significantly at elevated temperatures. Additionally, once opened, reformation of the cage-isomers is statistically highly unlikely. This makes the opening a nonreversible process. Therefore, these open-structure isomers are more favored when vibrational modes are excited by CID and are then kinetically trapped and detected following the subsequent rapid cooling.^45,46^ In addition, AIMD and IRC calculations confirm the possibility of structural transformation of the [B_12_I_7_]^−^ cluster from cage-like structure to open one during collisions (see Figures S7 and S8). Therefore, *n* = 7 is verified as a critical point of transformation during unwrapping of the B_12_ core. Upon these assignments, an excellent agreement between experimental and calculated first VDEs has been reached, with a mean absolute error (MAE) in VDE of only 0.069 eV (Table S1 and Figure S2).

**Figure 3:**
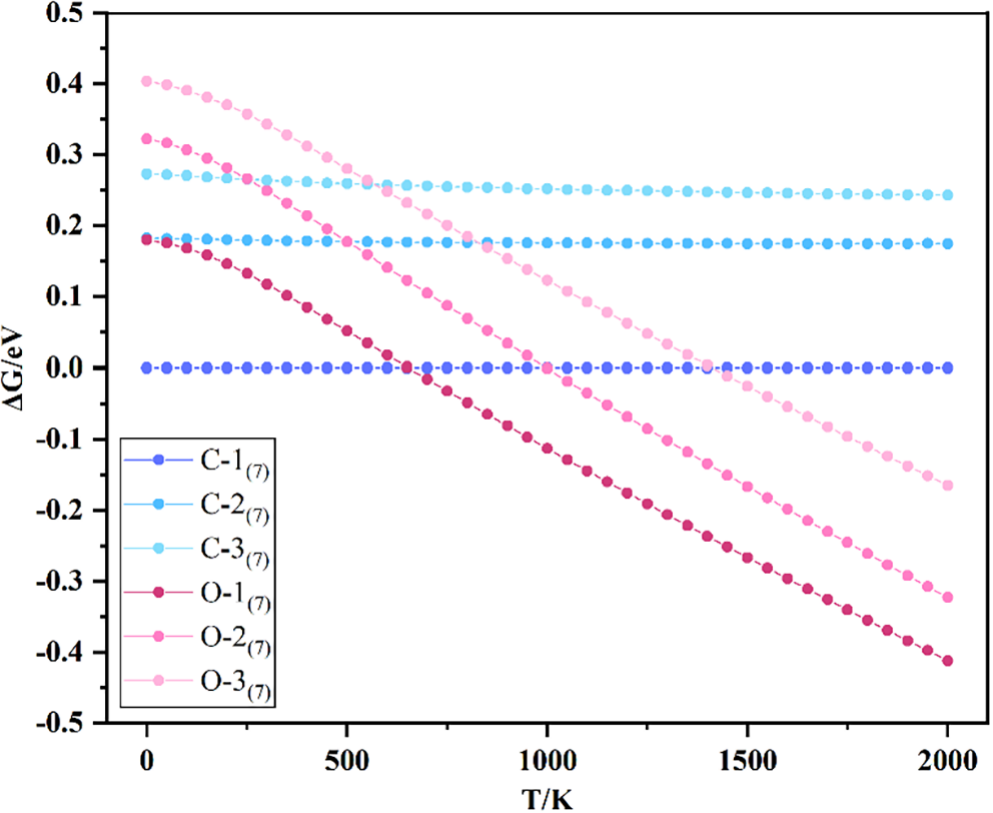
Temperature dependence of Δ*G* for the three lowest-free-energy isomers of the cage-like and open structures of [B_12_I_7_]^−^.

The energies of the highest (HOMO) or singly occupied molecular orbital (SOMO) for the respective [B_12_I_*n*_]^−^ anions with odd or even number of iodine ligands display a fluctuating decreasing trend that is consistent with the VDEs (see Figure S2). This indicates that the process of photodetaching these fragment anions follows the Koopmans’ theorem under single-particle transition framework.^47^ Comparing the spin density plots with the highest lying occupied orbital for [B_12_I_10_]^−^ (unrestricted calculations) indicates that the unpaired electron is not sitting in the highest occupied orbital but instead in α-HOMO–3. For all other radical ions, the spin-density is consistent with the highest lying (singly occupied) orbitals (SOMOs) as shown in Figure S9. The HOMOs or SOMOs for larger fragments [B_12_I_*n*_]^−^ (*n* = 12 to 8) consistently exhibit exclusive 5p-type electrons of iodine ligands. Notably, the HOMOs or SOMOs of further iodine stripped [B_12_I_*n*_]^−^ ions (*n* = 7 to 1) display significant delocalized multicenter bonding character in the boron core, showing either σ or π bonding characters. Interestingly, the B–B bond length diversifies after fragmentation of the [B_12_I_12_]^2–^ ion due to emergence of different vacant B atoms. The calculated averaged B–B Mayer bond order^48^ (MBO) values gradually increase from 0.53 to 0.85 as *n* decreases, indicating a more compact electronic structure with enhanced delocalization effects across the B–B bonds. Concurrently, the remaining B–I bonds become stronger with transition from weakly ionic type (0.23 of MBO) to covalent type (0.95 of MBO). As expected, the averaged bond lengths of B–B and B–I bonds are getting smaller and display a trend opposite to the MBOs (Figure S10). Adaptive natural density partitioning^49^ (AdNDP) analysis was performed for both the cage and open structures of [B_12_I_7_]^−^ (Figure S11). The results indicate that the cooperative delocalization of π and σ electrons contributes to the stability of the open structure.

The reactivity of [B_12_I_*n*_]^−^ ions with a quasi-icosahedral boron cage (*n* ≥ 8) has been previously discussed.^33,35,41^ These reactive ions were found to fill all vacant sites to achieve the stable, fully substituted [B_12_X_12_]-configuration. However, no reactivity investigations for [B_12_I_*n*_]^−^ with *n* ≤ 7 have been reported. Here, we examined the gas-phase reactions of all [B_12_I_*n*_]^−^ ions with allyl iodide. Allyl iodide is an established reagent for gas-phase ion–molecule reaction studies, offering various potential sites of attack for a reactive ion: a double bond, hydrogen atoms, and an iodine atom.^50–53^ The product ion mass spectra of all [B_12_I_*n*_]^−^ ions are shown in Figure 4a. For ions with an intact icosahedral boron cage (*n* = 11–8), the product spectra show two dominant products formed by the addition of the allyl radical, marked in green, and the addition of the entire allyl iodide molecule, marked in blue. Note that the loss of a hydrogen atom from the bound allyl radical or allyl iodide was observed in several cases. Since all vacant boron sites are reactive toward the organic reagent, hydrogen atom loss may be rationalized by multiple B–C bond formations. Also, the spin state of the corresponding ion plays a role: radical ions prefer to bind odd-electron groups. When the reaction time was 1 s, the [B_12_I_*n*_]^−^ (*n* = 11–8) ions reacted almost completely, as shown by their low abundance in the product spectrum (marked in gray). The reactivity significantly decreases for *n* < 8, as indicated by the high abundance of the [B_12_I_*n*_]^−^ (*n* = 7–1) precursor ions. In particular, for [B_12_I_7_]^−^, almost no product formation was observed and the precursor ion signal remained unchanged even for long reaction times, such as 10 s (Figure S12).

**Figure 4:**
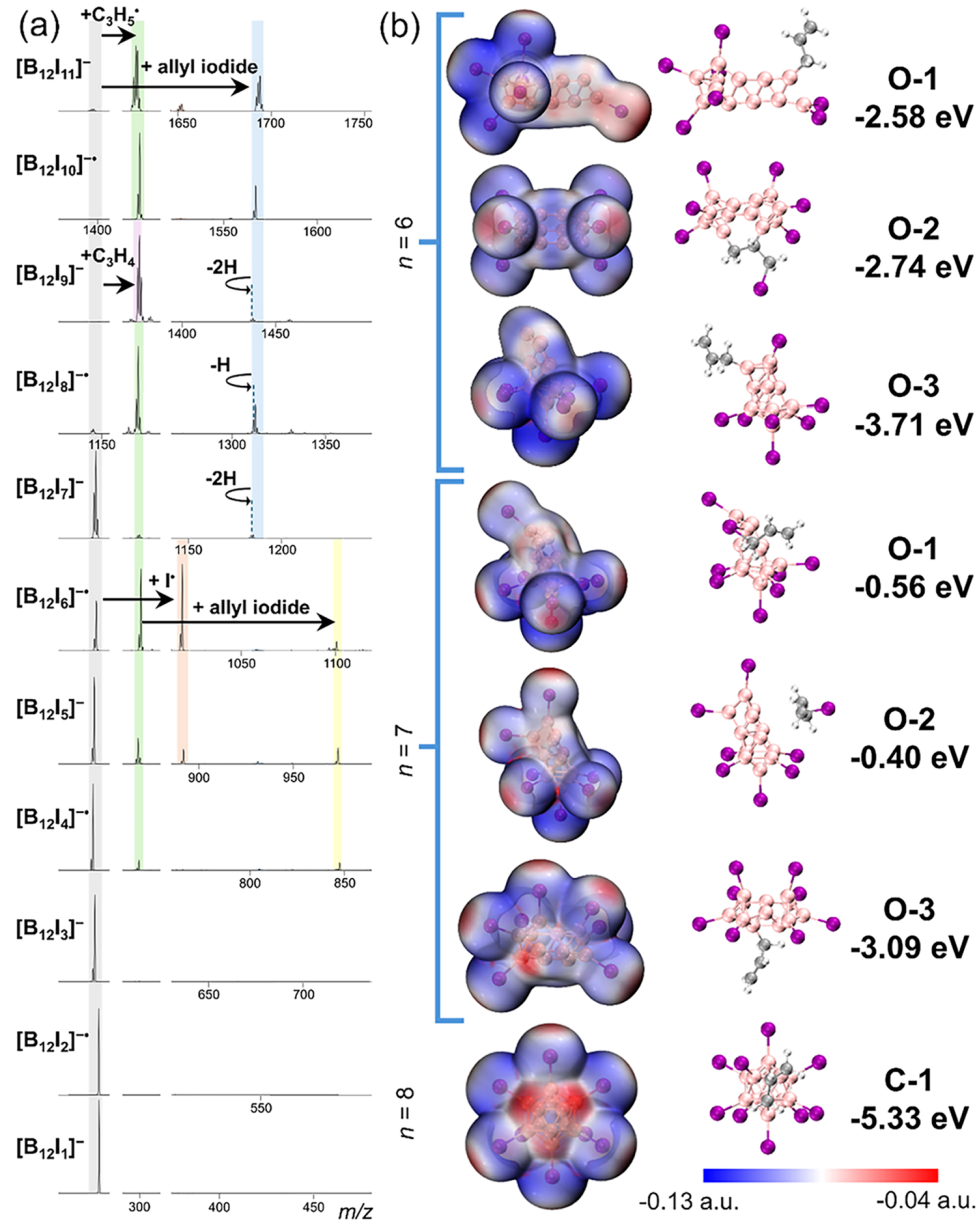
(a) Mass spectra obtained for isolated [B_12_I_*n*_]^−^ ions (*n* = 11 to 1, top to bottom) subjected to reactions with allyl iodide for one second in a linear quadrupole ion trap. Fast reactions involve the binding of an allyl moiety (green), of an entire allyl iodide moiety (blue), of both allyl and allyl iodide moieties (yellow), and of an iodine atom (orange). The isolated precursor ion is marked in gray. (b) Electrostatic potential (ESP) mapped onto an electron density isosurface (isovalue = 0.002) of [B_12_I_*n*_]^−^ (*n* = 6–8) and calculated reaction enthalpy (Δ*H*) for the interaction between [B_12_I_*n*_]^−^ clusters and C_3_H_5_I, computed at the ωB97X-2-D3­(BJ)/ma-def2-QZVPP//PBE0-D3­(BJ)/ma-def2-TZVP­(-f) level.

[B_12_I_6_]^−^, [B_12_I_5_]^−^, and [B_12_I_4_]^−^ exhibit increased reactivity compared to [B_12_I_7_]^−^ but are much less reactive than closed [B_12_I_*n*_]^−^ ions with *n* = 11–8. They also display a qualitatively different reaction behavior: direct binding of allyl iodide is no longer observed as a prominent reaction channel. Instead, the joint binding of the allyl radical and allyl iodide is observed (marked in yellow). Additionally, iodine atom abstraction becomes an important reaction channel for *n* = 6, 5 (marked in orange), which was only observed to occur very slowly for the ions with a quasi-icosahedral boron cage (*n* = 11–8). Ions with a planar boron structure (*n* < 4) do not form products of any significant abundance at a reaction time of 1 s. In summary, the reactivity of the [B_12_I_*n*_]^−^ ions toward allyl iodide changes stepwise during the structural transition from quasi-icosahedral (*n* = 11–8) to open (*n* = 7–4) to planar (*n* = 3–1). Interestingly, [B_12_I_7_]^−^ is less reactive than the smaller fragment ions of category 2. We assume that the main isomer of [B_12_I_7_]^−^ (presumably O-1_(7)_) is unreactive toward allyl iodide while a less abundant, more reactive isomer forms the detected minor adducts.

To explain the low reactivity of [B_12_I_7_]^−^, we calculated the electrostatic potential (ESP) distributions and reaction enthalpies (Δ*H*) for reactions with allyl iodide for the three lowest-energy open structures of [B_12_I_6_]^−^ and [B_12_I_7_]^−^ as well as the most stable structure of [B_12_I_8_]^−^ (Figure 4b). The results show that the energetically lowest lying open [B_12_I_7_]^−^ isomers (O-1_(7)_ and O-2_(7)_) exhibit vacant boron sites with weaker electrophilic character and significantly lower Δ*H* for allyl iodide binding than [B_12_I_8_]^−^ and the energetically low-lying [B_12_I_6_]^−^ isomers. This explains the reduced reactivity of [B_12_I_7_]^−^ toward nucleophilic allyl iodide. The O-3_(7)_ structure exhibits moderate electrophilic character at its exposed boron site and a Δ*H* similar to that of the [B_12_I_6_]^−^ isomers, suggesting that this isomer (present in the isomer mixture as indicated by NIPES data, see Figure 2) may be responsible for the low-abundance product ion formation observed for [B_12_I_7_]^−^ in Figure 4a. Furthermore, according to the complete ESP series for *n* = 11–1 (Figure S13), further transformation into planar structures (*n* = 3–1) leads to exposed boron sites with markedly reduced electrophilicity, which may explain why no reaction with allyl iodide was observed experimentally.

Since [B_12_I_*n*_]^−^ ions are the products of gas-phase reactions, mass-selected ion deposition experiments were used to investigate the reactivity of such ions in the condensed phase. This provides insights into their potential utilization in preparative methods.^54^ Deposition of mass-selected [B_12_I_*n*_]^−^ ions of category 1, in particular *n* = 11, has already been investigated in various studies.^35,41,55^ Similar to the observations made for gas-phase reactions, vacant boron atoms of the ions reacted with compounds on the surface, such as water (forming OH substituents), surface contaminants or intentionally provided reagents like organic molecules or peptides.^56,57^ Such partially dehalogenated clusters are interesting model systems for reactive intermediates formed by photochemical B–X bond cleavage. The photochemical substitution of halogens with OH groups was reported in 1968,^58^ and iodine–OH substitution was also observed in later photochemical reactions of [B_12_I_12_]^2–^.^7^

To gain insight into the reactions of the ions belonging to categories 2 and 3, 0.1 nmol of each [B_12_I_*n*_]^−^ ion (*n* = 7 to 3) were deposited in separate experiments on gold surfaces under comparable conditions using published methods.^35^ The surface layers were dissolved and analyzed using ESI-MS. Figure S14 shows the mass spectra and a color code visualizing the product distribution with respect to the number of iodine substituents and boron scaffold size. A list of all identified product anions is available in Table S4. Deposition of [B_12_I_*n*_]^−^ ions with *n* ≤ 7 leads to the formation of product mixtures of smaller *closo*-borate anions ([B_*m*_X_*m*_]^2–^ with *m* = 12, 11, 10, 8, 7, and 6 and X = H, OH, and I). Although singly charged ions were mass selected, the detected products are doubly charged, likely due to redox reactions at the conductive surface or by binding vacant electrophilic boron sites to available reaction partners at the surface by abstracting an anionic substituent (e.g., I^–^ or OH^–^). The lower stability of [B_12_I_6_]^−^ compared to [B_12_I_7_]^−^ and [B_12_I_5_]^−^ (based on CID and NIPES experiments) is also reflected in the product distribution found on the surface (detailed explanations are given in the Supporting Information). Absolute amounts of the detected products diminish with smaller *n*, indicating that stabilizing reactions forming dianionic *closo*-borate ions which can be detected in ESI-MS analysis are less probable for lower substitution levels—in particular for (quasi-)­planar ions.

## Conclusion

NIPE spectroscopy, ion mobility MS, gas-phase ion–molecule reactions, mass-selective ion deposition from the gas phase, and theoretical calculations were employed to systematically investigate the structural evolution, electronic properties, and reactivity of a series of [B_12_I_*n*_]^−^ clusters generated through the stepwise dissociation of the parent dianion [B_12_I_12_]^2–^ in the gas phase. The combined results demonstrate that these clusters undergo two major structural transitions upon sequential iodine cleavage, resulting in three major categories for the fragment ions: ions with cage-like structures (for *n* = 11–8), ions with open structures (for *n* = 7–4), and ions with planar (or quasi-planar) structures (for *n* = 3–1). The reactivity of [B_12_I_*n*_]^−^ ions decreases in a stepwise manner as their structures progressively evolve; however, [B_12_I_7_]^−^, as a critical turning point in the structural evolution, exhibits even lower reactivity than the less substituted ions with open structures (*n* = 6–4). This work complements the previously published picture of the transitions of B_12_ compounds during removal of substituents and closes the gap to understand the transition of icosahedral to quasi-planar B_12_ units. It also lays the basis for an in-depth understanding of structure, electronic structure and chemical reactivities of the rich variety of *closo*-borate anion fragments formed upon removal of multiple substituents from dodecaborate anions. Transferring these ions into the condensed phase further opens new possibilities for the generation of boron-containing compounds and materials by using dodecaborate fragment ions as building blocks, opening new possibilities to design boron-based molecular ions and materials of increasing importance in radiation medicine, energy storage, catalysis, and optoelectronic devices. Furthermore, although polyhedral boranes are generally considered to be highly stable, their opening and partial decomposition have recently attracted attention in synthetic approaches, such as electrochemical decomposition and C–H borylation using strong boron electrophiles.^59,60^ Therefore, the combined gas phase approach of photoelectron spectroscopy, ion mobility, computational predictions, gas phase ion chemistry and preparative mass spectrometry of various borate anions may help to shed more light on intermediate structures formed in more common synthetic approaches including photochemistry and electrochemistry.

## Experimental and Theoretical Methodologies

### NIPE Spectra Measurement

The parent *closo*-dodecaborate [B_12_H_12_]^2–^ anions were prepared according to a literature procedure.^61^ Iodination of Na_2_[B_12_H_12_] following literature methods yielded the corresponding iodinated [B_12_I_12_]^2–^.^62^ Ammonium salts were precipitated by adding ammonium chlorides into aqueous solutions of the sodium salts. The equipment used in the PNNL NIPES experiments consisted of an electrospray ionization source (ESI), a cryogenic ion trap, and a magnetic bottle time-of-flight (TOF) photoelectron spectrometer, featuring the ability of size selection.^63^ The [B_12_I_*n*_]^−^ anions were generated by electrospraying a 1 mM acetonitrile solution of (NH_4_)_2_[B_12_I_12_] with blowing N_2_ gas toward the spray tip to enable in-source CID. They were then transmitted from the ESI interface region into the vacuum using a radio frequency quadrupole ion guide and were detected with a downstream quadrupole mass spectrometer to ensure optimal CID conditions and stability of the ion cluster beams. The anions were subsequently directed through a 90° bender into a cryogenic ion trap, where they were accumulated and cooled to 20 K by collisions with a cold buffer gas (pure helium) for approximately 20–100 ms. After cooling, the anions were pulsed into the extraction zone of the TOF mass spectrometer, where ions were separated based on their mass-to-charge ratios at a repetition rate of 10 Hz. In each NIPES experiment, the targeted [B_12_I_*n*_]^−^ clusters, after mass selection and maximal deceleration, were photodetached using a 157 nm (7.867 eV) F_2_ laser. This laser operated at a frequency of 20 Hz. The anion beam was alternately turned off to achieve shot-to-shot background subtraction. The released photoelectrons were collected by the magnetic bottle with an efficiency close to 100%. The TOF analysis of the photoelectrons was performed using a calibrated electron flight tube with an energy resolution of 2%. After calibration with the known spectra of I^–^ and Au­(CN)_2_^–^, the electron binding energy (EBE) was obtained by subtracting the electron kinetic energy from the photon energy of detachment.

### Ion Mobility Spectroscopy

[B_12_I_12_]^2–^ ammonium salts (<1 mg) were dissolved in 5 mL acetonitrile (ulc grade, Biosolve BV, Netherlands) and diluted further 1:10 with acetonitrile. The diluted solution was infused at a flow rate of 5 μL/min into an ESI source connected to a Synapt G2 *Si* instrument (Waters Corporation, Manchester, U.K.) by using the following source settings: negative polarity resolution mode, 3 kV capillary voltage, 100 °C source temperature, 30 V cone voltage, source offset 80, 250 °C desolvation temperature, 20 L/h cone nitrogen gas flow and 600 L/h desolvation nitrogen gas flow. IMS settings were 600 m/s wave velocity and 40 V wave height. Polyalanine was used to calibrate IMS. Spectra were recorded using a *m*/*z* range from 50 up to 2000. MS/MS experiments were carried out by either setting trap collision cell voltage to a fixed value (0, 20, 25, 30, 40, 50, 60, 70, 80, 90, 100, 120, 140, 160, or 200 V) or by ramping the collision cell voltage from 15 to 200 V. Data analysis was performed with MassLynx (version 4.2 SCN983) and Drift Scope (version 2.9).

### Quantum Chemical Calculations

To obtain the global minimum and low-lying isomers of [B_12_I_*n*_]^−^ clusters, a theoretical strategy by combining the “top-down” stepwise “peeling” and “bottom-up” atomic recombination is proposed. The “top-down” stepwise “peeling” method starts with the parent [B_12_I_12_]^2–^ cluster. A comprehensive set of initial configurations for [B_12_I_*n*_]^−^ (*n* = 11–1) was generated by exhaustively considering all possible scenarios for the detachment of I^–^ or I^•^. The generated initial structures were optimized at the PBE0 ^64^/ma-def2-TZVP­(-f)^65^ level with Grimme’s dispersion corrections of D3­(BJ)^66,67^ by using Gaussian 16 software.^68^ The second “bottom-up” atomic recombination method is also known as an effective global structural optimization tool. Approximately > 100,000 initial structures were randomly generated by genmer code^69^ through iterative sampling and followed by subsequent semiempirical GFN2-xTB^70,71^ optimizations using CREST code.^72^ Single-point (SP) energy calculations were performed at the r^2^SCAN-3c^73^ level to ensure that the low-lying structures selected had relative energies (RE) below 10 kcal/mol. The structures obtained using both methods were submitted to identical subsequent processing; the resulting structures were reoptimized at the PBE0-D3­(BJ)/aug-cc-pVTZ­(-pp)^74,75^ level. Simultaneously, harmonic vibrational frequency analyses were performed at the same level to ensure that the identified structures correspond to minima on the potential energy surfaces and had no imaginary frequencies. The final SP energies were calculated at the high-level DLPNO–CCSD­(T)^76^/aug-cc-pVTZ­(-pp) method using the ORCA 5.0.4 code.^77^ The unrestricted Kohn–Sham (UKS) wave function was alternatively used to allow a more accurate description for partial open-shell structures and the final SP energies were calculated using the (U)­KS-DLPNO-CCSD­(T) approach based on the BP86 functional.^78,79^ The thermal corrections of Gibbs free energies were calculated at the PBE0/aug-cc-pVTZ­(-pp) level using the Shermo code^80^ with a zero-point energy scale factor of 0.9771 ^81^ and the thermally corrected values at 20 K were subsequently evaluated to determine the most thermodynamically stable configuration. The vertical detachment energies (VDEs) were calculated based on the optimized geometries of the dianions at the same level, by evaluating the SP energy differences between the corresponding monoanions and dianions. The density of states (DOS), highest occupied molecular orbitals (HOMOs), singly occupied molecular orbitals (SOMOs), lowest unoccupied molecular orbitals (LUMOs), spin density, the adaptive natural density partitioning (AdNDP) analysis and electrostatic potential (ESP) were computed using the Multiwfn code^82^ at the same level as optimization, with the corresponding isosurfaces visualized through the VMD program.^83^ The analysis of Mayer bond order for B–B and B–I bonds were performed at the PBE0/cc-pVTZ­(-pp) level using the Multiwfn code. Ab initio molecular dynamics (AIMD) simulations were conducted using the ORCA 5.0.4 code at the BHandHLYP^84^-D3­(BJ)/ma-def2-SVP^65^ level. The time step for the simulations was set to 0.5 fs, resulting in a total simulation trajectory of 1 ps. Additionally, potential energy surface calculations based on transition state theory were performed to provide a detailed examination of the interconversion process between the isomers. Transition state optimization was performed at the PBE0-D3­(BJ)/def2-SVP level,^85^ followed by an intrinsic reaction coordinate (IRC) calculation with energy barriers refined at the DLPNO-CCSD­(T)/aug-cc-pVTZ­(-pp) level. The reactions between [B_12_I_*n*_]^−^ and allyl iodide were studied theoretically, with geometry optimizations performed at the PBE0-D3­(BJ)/ma-def2-TZVP­(-f) level and single-point energy calculations carried out at the ωB97X-2-D3­(BJ)/ma-def2-QZVPP level. Thermal corrections to the enthalpies at 298.15 K were calculated at the PBE0-D3­(BJ)/ma-def2-TZVP­(-f) level using the Shermo code. CCS values were computed using IMoS^86^ with parameters for the calculation shown in Table S3a. All outputs of relevant calculations are published in Zenodo and can be retrieved via the following link: 10.5281/zenodo.18821969

### Gas-Phase Ion–Molecule Reactions

All mass spectrometry experiments were conducted using a modified Thermo Scientific LTQ linear quadrupole ion trap (LQIT) mass spectrometer equipped with an electrospray ionization (ESI) source.^87^ The analyte solutions were prepared in methanol at 1 mM concentration and were directly introduced into the ESI source at a flow rate of 10 μL/min by using a 500 μL Hamilton syringe. The ESI source conditions used were 3 kV spray voltage, 20–30 (vendor-specific arbitrary units) of sheath gas (N_2_) flow rate, 10 (vendor-specific arbitrary units) of auxiliary gas (N_2_) flow rate, and 275 °C capillary temperature. After ionization of the analytes, the ions were transferred into the ion trap, isolated using an isolation width of 0.5–2.0 *m*/*z*-units and a *q* value of 0.25 and were allowed to react with allyl iodide for up to 1,000 ms before all ions were ejected for detection. The reagent, allyl iodide, was introduced into the ion trap (containing 2 mTorr of helium buffer gas) via an external reagent mixing manifold using a syringe drive at a flow rate of 2 μL/h and diluted with helium before entering the ion trap through a variable leak valve. For collision-induced dissociation (CID) experiments, the advanced scan features of the LTQ Tune Plus interface were used to isolate the ions by using an *m*/*z* window of 2 units. At a *q* value of 0.25, the ions were subjected to CID (collision energy 20–50 arbitrary units) for 30 ms by using helium as the collision gas. All mass spectra acquired were an average of at least 20 individual mass spectra. Xcalibur 2.0 software was used to process the data.

### Fragment Ion Deposition

Deposition of [B_12_I_*n*_]^−^ (*n* = 3–7) was performed in a previously described ion soft-landing instrument optimized for fragment ion deposition.^35^ In individual deposition experiments, 100 pmol of each fragment ion were mass-selected and deposited on gold surfaces. The deposition was carried out in a controlled NO atmosphere at a pressure of *p* = 5–7 × 10^–5^ mbar. After deposition, the gold surfaces were removed from the instrument, and the deposited substance was dissolved in acetonitrile and analyzed using (−)­ESI-HRMS with an Exploris 480 mass spectrometer (Thermo Fisher Scientific, Bremen, Germany) in negative ion mode. Further details on the deposition process can be found in the Supporting Information, Table S5. Results from kinetic energy measurements of the deposited ions are shown in the Supporting Information, Figures S16–S20.

## Supplementary Material



## Notes

Quadrupole and HRMS spectra of [B_12_I_*n*_]^−^ (*n* = 1–11); experimental and calculated first VDEs and the corresponding frontier orbitals of [B_12_I_*n*_]^−^ (*n* = 1–11); experimental first VDEs (eV) of [B_12_I_*n*_]^−^ clusters from NIPES and theoretical VDEs calculated at the DLPNO–CCSD­(T)/aug-cc-pVTZ­(-pp) level, and mean absolute errors (MAEs); ions observed during IMS measurements with assignments, TWIMS collision cross sections (TWCCS) determined from three independent measurements (M1–M3) for each signal; the workflow of the systematic optimization protocol performed in this work; optimized structures of the global minimum and low-lying isomers of [B_12_I_*n*_]^−^ (*n* = 1–11) calculated at the PBE0-D3­(BJ)/aug-cc-pVTZ­(-pp) level; methods and parameters used for CCS calculations with IMoS, and computed CCS values (with linear scaling) for [B_12_I_*n*_]^−^ (*n* = 1–11); a series of [B_12_I_6_]^−^ isomers were generated from O-1(7) by iodine removal, with Δ*G* referenced to O-1(6); comparison of [B_12_I_*n*_]^−^ IMS data (*n* = 5, 4 vs 10, 9); AIMD simulations at the BHandHLYP-D3­(BJ)/ma-def2-SVP level and IRC-based TS calculations at the PBE0-D3­(BJ)/def2-SVP level, with DLPNO–CCSD­(T)/aug-cc-pVTZ­(-pp) single-point refinements, were used to explore the potential energy surfaces and isomerization pathways of [B_12_I_7_]^−^; IRC curves for the transition-state isomerization of [B_12_I_7_]^−^ calculated at the PBE0-D3­(BJ)/def2-SVP level; SOMOs and spin density of open-shell [B_12_I_*n*_]^−^ clusters (even *n*); calculated MBOs and bond lengths of B–B and B–I bonds for selected [B_12_I_*n*_]^−^ clusters; AdNDP plots for the coexisted isomers of [B_12_I_7_]^−^ at the PBE0-D3­(BJ)/aug-cc-pVTZ­(-pp) level; mass spectrum of isolated [B_12_I_7_]^−^ after 10 s reaction with allyl iodide; a color code visualizing the product distribution of deposited [B_12_I_n_]^−^ (*n* = 7–3) ions with respect to the number of iodine substituents and boron scaffold size; HRMS spectrum of product anions after fragment ion deposition of [B_12_I_*n*_]^−^ (*n* = 3–7); example of background subtraction applied to MS spectra after dissolution of surface layers formed by fragment ion deposition of [B_12_I_*n*_]^−^ (*n* = 3); electrostatic potential (ESP) plotted on the molecular surface of [B_12_I_*n*_]^−^ (*n* = 11–1); settings/conditions applied during fragment ion deposition; ion current of [B_12_I_*n*_]^−^ (*n* = 3–7) as a function of retarding potential with sigmoidal fitting, and the corresponding probability density of kinetic energy per charge derived from the fit (PDF)
