# Supplementary material for: Unwrapping the Dodecaborane Core: Structure, Electronic Properties, and Chemical Reactivity Across the Complete [B12I n ]− Series (n = 11–1)
Source: J Am Chem Soc. 2026 May 21;148(21):22166–77. doi: 10.1021/jacs.6c04725 (PMC13244469; doi:10.1021/jacs.6c04725)
Supplement: Supplementary file 1 [file ja6c04725_si_001.pdf]

SUPPORTING INFORMATION FOR

Unwrapping the Dodecaborane Core: Structure, Electronic Properties, and Chemical Reactivity Across the Complete $[B_{12}I_n]^-$ Series (n = 11–1)

Qiaoqiao Shao[1], Wenjin Cao[2], Harald Knorke[3], Kay Antonio Behrend[3], Jaskiran Kaur[4], Markus Rohdenburg[3], Daniela Volke[5], Hilkka Kenttämaa[4], Zhubin Hu[1], Zhenrong Sun[1], Jonas Warneke[3,6*], Haitao Sun[1,7*] and Xue-Bin Wang[2*]

[1] *State Key Laboratory of Precision Spectroscopy, School of Physics, East China Normal University, Shanghai 200241, China*

[2] *Physical Sciences Division, Pacific Northwest National Laboratory, 902 Battelle Boulevard, P.O. Box 999, Richland, Washington 99352, USA*

[3] *Wilhelm-Ostwald-Institut für Physikalische und Theoretische Chemie, Universität Leipzig, Linnéstr. 2, 04103 Leipzig, Germany*

[4] *James Tarpo Jr. and Margaret Tarpo Department of Chemistry, Purdue University, West Lafayette, 560 Oval Drive, IN, 47907, USA.*

[5] *Institute of Bioanalytical Chemistry, Faculty of Chemistry and Center for Biotechnology and Biomedicine, Universität Leipzig, 04103 Leipzig, Germany*

[6] *Leibniz-Institut für Oberflächenmodifizierung e.V. (IOM), 04318 Leipzig, Germany*

[7] *Collaborative Innovation Center of Extreme Optics, Shanxi University, Taiyuan, Shanxi 030006, China*

*Corresponding author: jonas.warneke@uni-leipzig.de (J.W.); htsun@phy.ecnu.edu.cn (H.S.) and xuebin.wang@pnnl.gov (X.-B.W.).

a)

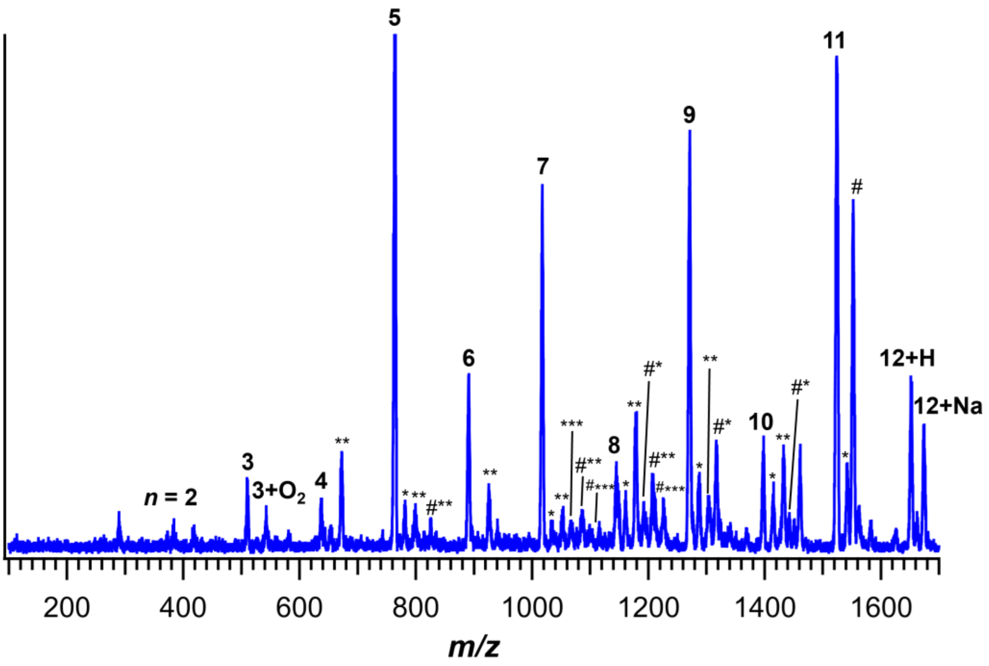

b)

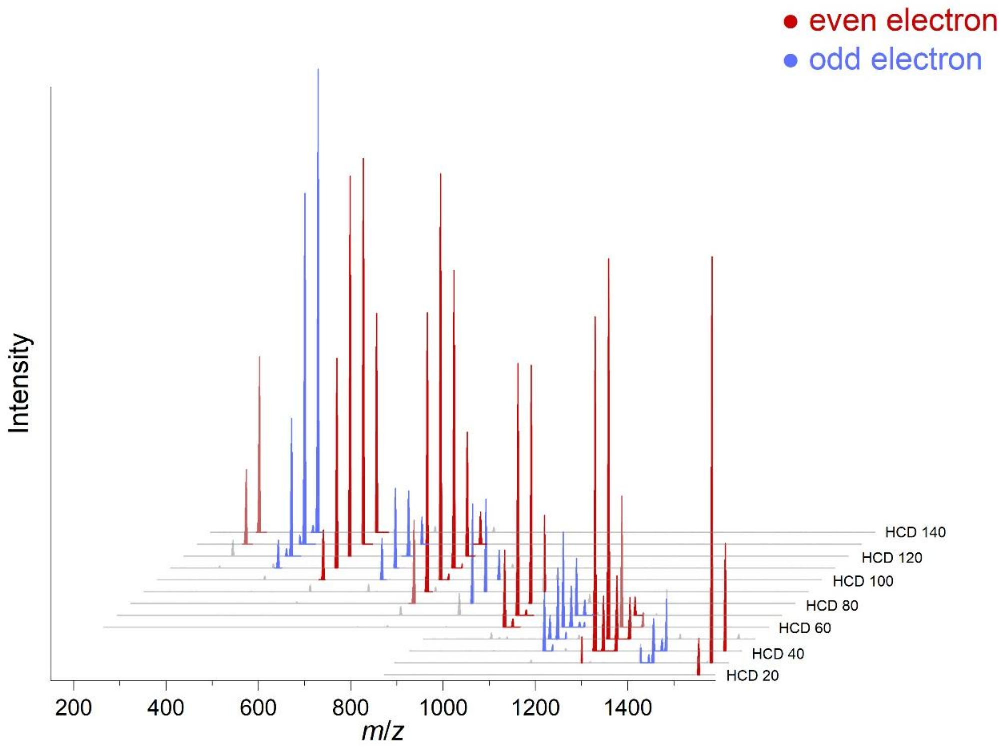

Figure S1. (a) Quadrupole mass spectra of $[B_{12}I_n]^-$ (n = 1 – 11) via in-source CID by electrospraying 1mM $(NH_4)_2[B_{12}I_{12}]$ acetonitrile solution using the PNNL NIPES instrument. An initial assignment for coupling peaks using the style in Fig1 ("#" for $+N_2$, "*" for $+H_2O/OH$). Most of the detected ions are consistent with the ions measured

in the ion mobility instrument (see Fig.1). (b) HR-MS spectra (Exploris 480, Thermo Scientific). A solution of $K_2[B_{12}I_{12}]$ in acetonitrile was diluted to a concentration of 10^{-8} M and injected at a flow rate of 2 μL min^{-1}. An ESI spray voltage of −2.6 kV and an ion transfer tube temperature of 300 C were used. For fragmentation, higher-energy collision-induced dissociation (HCD) conditions were employed using N_2 as collision gas. Normalized collision energies are shown in manufacturer-specified arbitrary units. For lower HCD energies (20-60), the m/z range typically showing doubly negatively charged ions is removed for the sake of visual clarity. Fragment ions with an even electron shell are highlighted in red, while odd electron shell fragment ions are highlighted in blue. Note that fragment ions can be subject to spontaneous residual gas addition after fragmentation in the HCD cell.

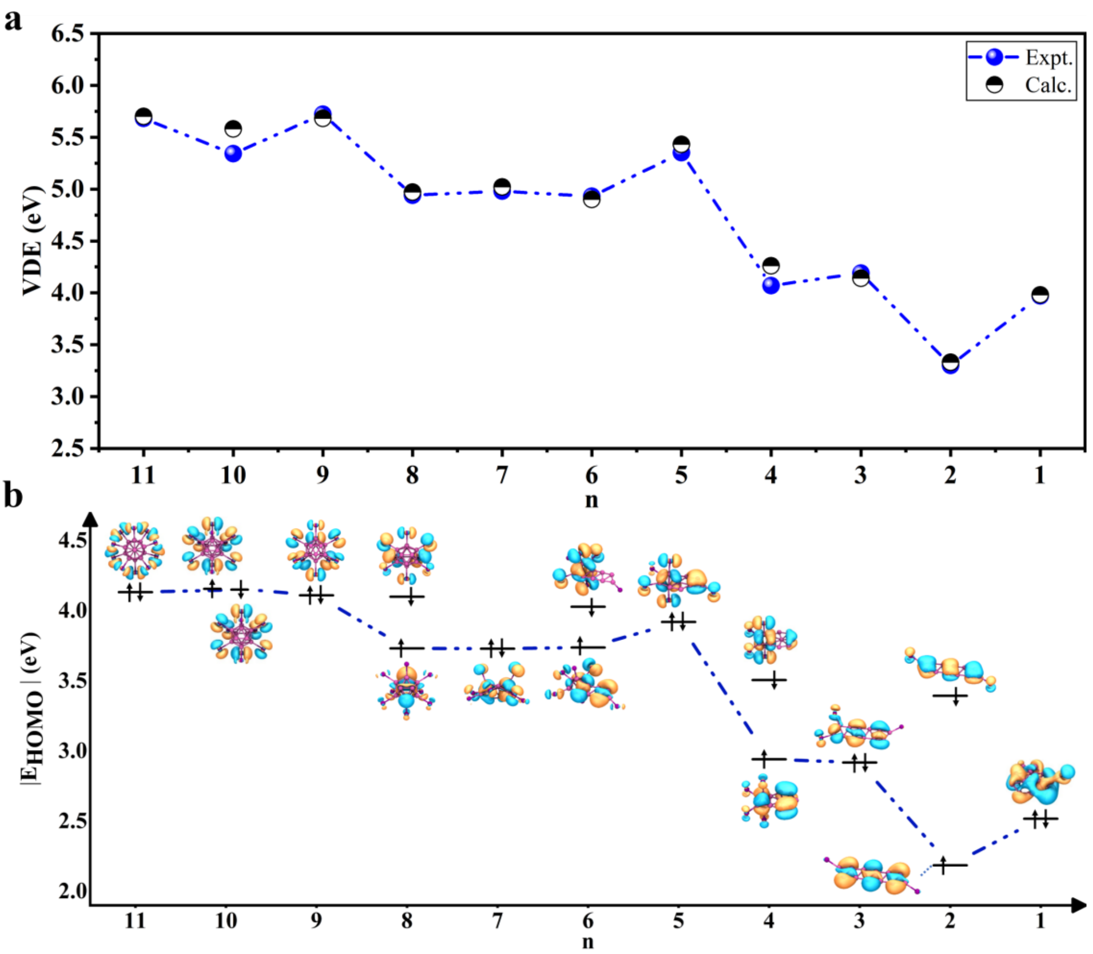

Figure S2 (a) Experimental 1st VDEs, determined from the lowest eBE band from each spectrum, in comparison to the calculated VDEs based on the identified isomers contributed to the experiments for $[B_{12}I_n]^-$ (n = 11 – 1) clusters. (b) The corresponding highest occupied molecular orbitals (HOMOs) for odd n or singly occupied molecular orbitals (SOMOs) of alpha (up arrow) and beta (down arrow) spin electrons for even n of $[B_{12}I_n]^-$ plotted with an isovalue of 0.03.

Table S1. Experimental 1st VDEs (in eV) of $[B_{12}I_n]^-$ clusters by NIPES measurements and theoretical VDEs calculated at the DLPNO-CCSD(T)/aug-cc-pVTZ(-pp) level based on the isomeric structures identified contributing to the experiments. The mean absolute errors (MAEs) between experimental and theoretical VDEs are also listed.

**VDE**	$[B_{12}I_n]^-$ (n = 11 – 1)										
**n**	11	10	9	8	7	6	5	4	3	2	1
**Expt.**[a]	5.68	5.34	5.72	4.94	4.98	4.93	5.35	4.07	4.19	3.30	3.97
**Calc.**	5.70	5.58	5.68	4.97	5.02[b]	4.90	5.43	4.26	4.14[c]	3.33	3.98
**C-E**	0.02	0.24	-0.04	0.03	0.04	-0.03	0.08	0.19	-0.05	0.03	0.01
**MAE**	0.069										

[a] Expt. error is ± 0.02 for n = 11, 9, 7, 6, 5, 4, 3, 2, 1 where a sharp 1st resolved peak is well defined, while ± 0.05 for n = 10, 8 where the 1st band is relatively broad.

[b] Open-3 isomer (n = 7). [c] Open-2 isomer (n = 3).

Table S2. Ions observed during IMS measurements with assignments. For each signal, three individual measurements M1-M3 were used to determine the TWIMS collisional cross section ^{TW}CCS.

Ions	*m/z*	M1 $^{TW}CCS_{N2}$	M2 $^{TW}CCS_{N2}$	M3 $^{TW}CCS_{N2}$	M1 dt [bins]	M2 dt [bins]	M3 dt [bins]	M1 dt [ms]	M2 dt [ms]	M3 dt [ms]
$[B_{12}I_{12}]^{2-}$	827.48	241.9	241.2	241.5	26.5	26.4	26.4	1.9	1.9	1.9
$[B_{12}I_{12}]^{2-}$	827.48	274.2	274.0	274.1	32.7	32.7	32.7	2.3	2.3	2.3
$[B_{12}I_{11}]^{2-}$	764.03	213.2	*	211.8	21.4	*	21.2	1.5	*	1.5
$[B_{12}I_{11}]^{2-}$	764.03	233.7	233.4	233.5	25.0	24.9	24.9	1.7	1.7	1.7
$[B_{12}I_{10}]^{2-}$	700.58	203.1	203.7	203.3	19.6	19.7	19.6	1.4	1.4	1.4
$[B_{12}I_{10}]^{2-}$	700.58	229.4	228.9	229.1	24.2	24.1	24.1	1.7	1.7	1.7
$[B_{12}I_{11}N_2]^-$	1556.07	209.1	209.3	209.2	67.4	67.5	67.4	4.6	4.6	4.6
$[B_{12}I_{11}]^-$	1528.06	209.1	209.2	208.9	67.3	67.4	67.2	4.7	4.7	4.7
$[B_{12}I_{10}N_2]^-$	1429.16	202.3	202.2	202.2	63.6	63.6	63.5	4.3	4.3	4.3
$[B_{12}I_{10}H(OH)]^-$	1419.17	199.9	200.1	200.0	62.3	62.4	62.4	4.3	4.3	4.3
$[B_{12}I_{10}]^-$	1401.16	202.2	202.1	202.2	63.5	63.5	63.5	4.3	4.3	4.3
$[B_{12}I_9N_2H(OH)]^-$	1320.27	197.8	198.1	198.0	61.2	61.3	61.2	4.2	4.2	4.2
$[B_{12}I_9H_2(OH)_2]^-$	1310.27	*	196.7	*	*	60.5	*	*	4.1	*
$[B_{12}I_9H(OH)]^-$	1292.26	192.8	192.9	192.8	58.4	58.4	58.4	4.0	4.0	4.0
$[B_{12}I_9]^-$	1274.25	191.3	191.3	191.3	57.6	57.6	57.6	3.9	3.9	3.9
$[B_{12}I_8N_2H(OH)_2]^-$	1210.37	193.0	193.5	193.2	58.4	58.6	58.5	4.0	4.0	4.0
$[B_{12}I_8N_2H(OH)]^-$	1193.36	190.0	190.1	190.1	56.9	56.9	56.9	3.9	3.9	3.9
$[B_{12}I_8H_2(OH)_2]^-$	1183.37	188.5	188.6	188.5	56.1	56.1	56.1	3.8	3.8	3.8
$[B_{12}I_8H(OH)]^-$	1165.36	185.3	185.2	185.2	54.5	54.4	54.4	3.7	3.7	3.7
$[B_{12}I_8]^-$	1147.35	183.6	183.5	183.5	53.6	53.6	53.5	3.7	3.7	3.7
$[B_{12}I_7N_2H(OH)_3]^-$	1100.47	186.0	186.1	186.1	54.7	54.8	54.8	3.7	3.7	3.7
$[B_{12}I_7N_2H_2(OH)_2]^-$	1084.47	185.0	185.2	185.0	54.2	54.3	54.2	3.7	3.7	3.7
$[B_{12}I_7H_2(OH)_3]^-$	1073.47	183.0	182.8	182.8	53.2	53.1	53.1	3.6	3.6	3.6
$[B_{12}I_7H(OH)_2]^-$	1055.46	180.5	180.3	108.4	52.0	51.9	51.9	3.6	3.5	3.5
$[B_{12}I_7H(OH)]^-$	1038.45	179.2	179.0	179.2	51.3	51.2	51.3	3.5	3.5	3.5
$[B_{12}I_7]^-$	1020.44	188.9	189.2	189.0	56.1	56.2	56.1	3.8	3.8	3.8
$[B_{12}I_7]^-$	1020.44	202.2	202.5	202.3	63.0	63.2	63.1	4.3	4.3	4.3
$[B_{12}I_6]^-$	893.54	179.9	180.0	179.9	51.4	51.5	51.4	3.5	3.5	3.5
$[B_{12}I_6]^-$	893.54	189.8	190.1	189.9	56.3	56.4	56.3	3.9	3.9	3.9
$[B_{12}I_5]^-$	766.63	180.9	180.9	180.9	51.7	51.7	51.6	3.5	3.5	3.5
$[B_{12}I_4]^-$	639.73	163.4	163.4	163.3	43.0	43.1	43.0	2.9	2.9	2.9
$[B_{12}I_3]^-$	512.83	151.1	150.9	150.8	37.4	37.3	37.2	2.6	2.5	2.5
$[B_{12}I_3]^-$	512.83	161.9	161.9	161.9	42.0	42.0	42.0	2.9	2.9	2.9
$[B_{12}I_2]^-$	385.92	147.6	147.6	147.6	35.4	35.5	35.3	2.4	2.4	2.4
$[B_{12}I_1]^-$	259.02	128.1	128.1	128.1	26.9	26.9	26.9	1.8	1.8	1.8

* not detected

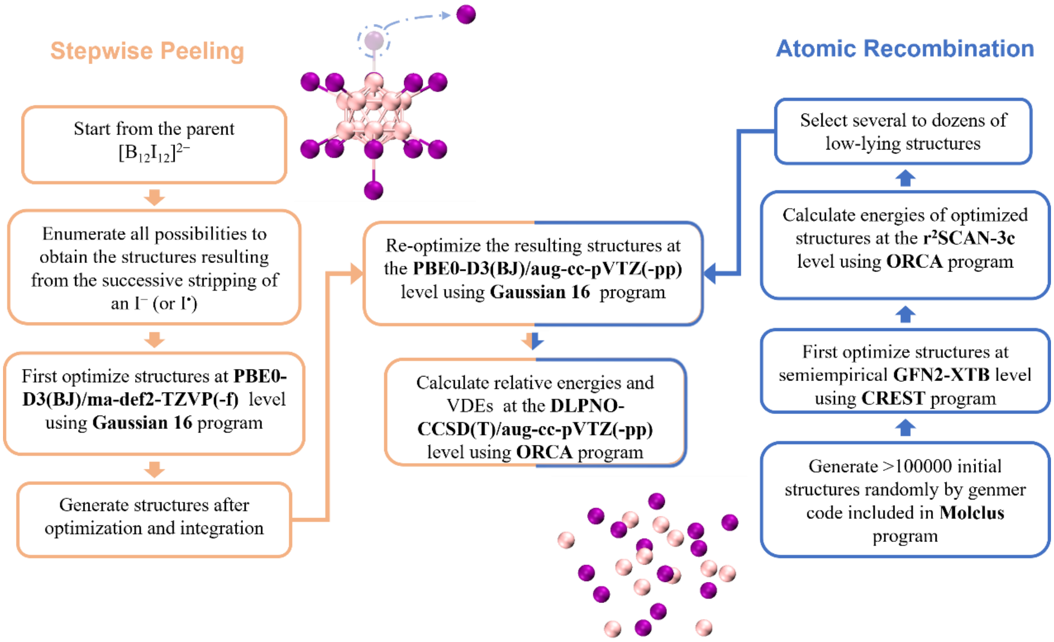

Figure S3 The workflow of the systematic optimization protocol performed in this work. The "top-down" stepwise peeling (left) and the "bottom-up" atomic recombination strategy (right) are combined to obtain the global minimum and low-lying isomers of $[B_{12}I_n]^-$ clusters.

First, the "top-down" stepwise peeling strategy simulates dissociation process by starting from the parent molecule and sequentially removing I^- (or $I^•$) one by one. In the early stage of sequential dissociation with only fewer iodine ligands stripped away, such a method can effectively identify the most stable cage-like structures, based on which the predicted VDEs are in good agreement with corresponding experimental data. As more iodine ligands are stripped away, the limitation of this strategy appears accompanied with the predicted structures remaining in a cage-like configurations without structural deformation. As a result, the corresponding computed VDEs show significant discrepancies compared to the experimental values. To overcome this, an alternative "bottom-up" atomic recombination method is introduced. This method involves extensive random recombination of all atoms to achieve a more comprehensive conformational search for the global minimum, thereby identifying possible low-lying isomers besides the cage-like structures. And the "bottom-up" method through conformational searching is proved to be robust particularly when a greater number of iodine atoms were removed.

While the $[B_{12}I_n]^-$ series was previously investigated by the Heine group in 2012[1], a more rigorous and comprehensive computational re-examination is essential for several reasons. Firstly, the complex structural evolution of the B_{12} core—transitioning from an icosahedron to a planar geometry—requires a more exhaustive sampling of the potential energy surface (PES) to ensure the identification of the true global minima. Secondly, to achieve a high-fidelity correlation with the high-resolution NIPE and IMS data, electronic energies and structural parameters must be determined at a higher level of theory than the methods employed a decade ago.

Our approach significantly improves upon the 2012 study[1] in both configuration search of clusters and chemical accuracy. First, we implemented a dual-pathway structural search: a "top-down" stepwise peeling and a "bottom-up" stochastic recombination using the Molclus software. This resulted in over 100,000 initial configurations, ensuring a much higher probability of capturing low-lying isomers that might have been overlooked previously. Second, the computational framework employed in the 2012 study[1], while pioneering at the time, relied on relatively low-level density functional theory (DFT) methods. Specifically, their structural optimizations were performed using the BP86 pure functional with TZP basis sets, and single-point (SP) energies were calculated at the B3LYP/TZP level. However, these methods have limitations in accurately capturing the subtle energetic differences and dispersion interactions critical for large, substituted boron clusters like $[B_{12}I_n]^-$. In our work, we have significantly elevated the level of theory to ensure higher reliability: we utilized the PBE0-D3(BJ) functional with large augmented basis sets (aug-cc-pVTZ) for optimizations to properly account for electronic exchange-correlation and long-range dispersion. More importantly, we refined our final energies using the DLPNO-CCSD(T) method—the "gold standard" in quantum chemistry—. This transition from standard DFT to high-level ab initio single-point calculations provides a more rigorous energetic landscape, which is indispensable for the accurate assignment of NIPE spectra and the interpretation of the IMS-observed structural transitions.

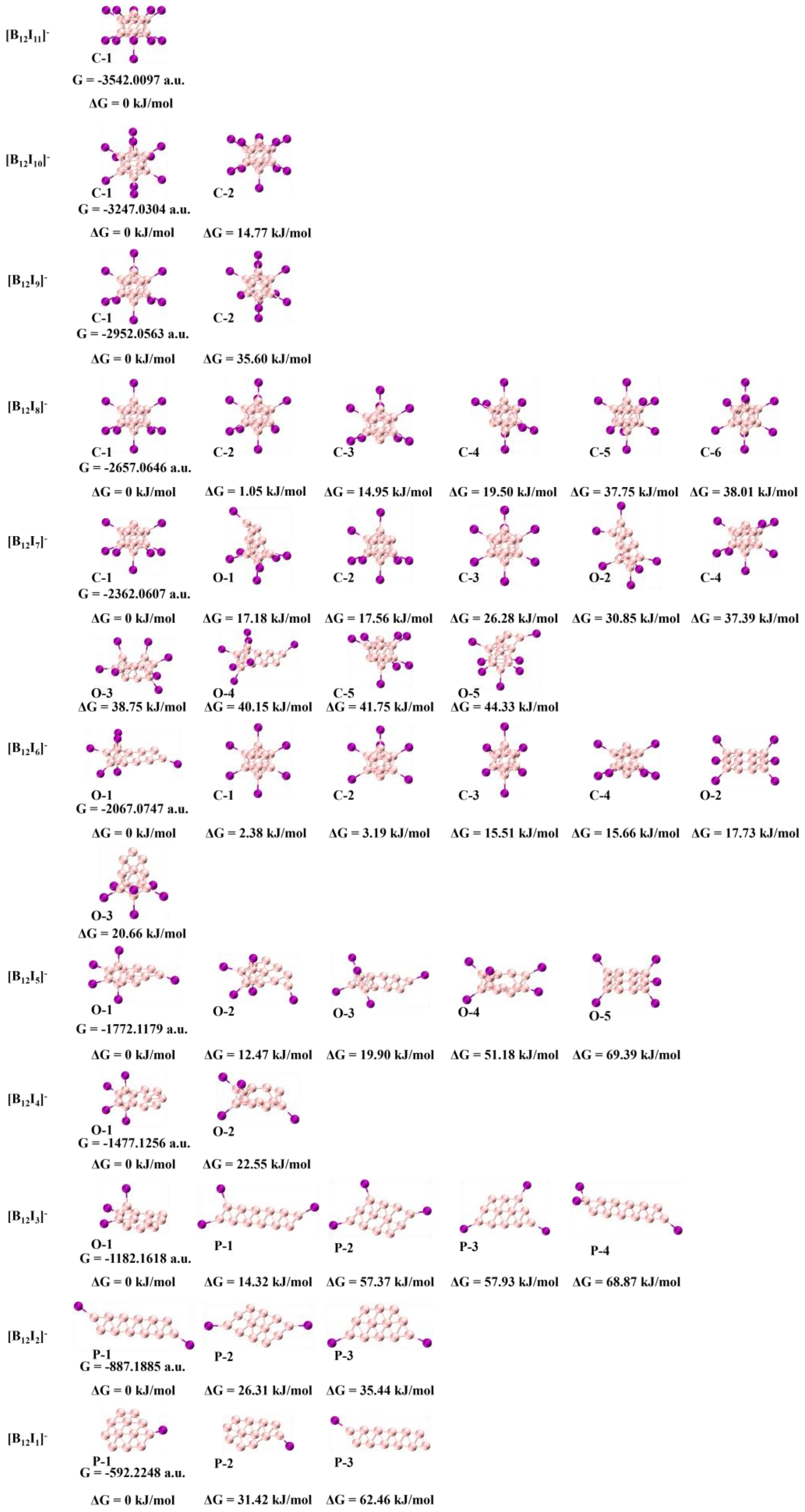

[B12I11]-
C-1
G = -3542.0097 a.u.
ΔG = 0 kJ/mol
[B12I10]-
C-1
C-2
G = -3247.0304 a.u.
ΔG = 0 kJ/mol
ΔG = 14.77 kJ/mol
[B12I9]-
C-1
C-2
G = -2952.0563 a.u.
ΔG = 0 kJ/mol
ΔG = 35.60 kJ/mol
[B12I8]-
C-1
C-2
C-3
C-4
C-5
C-6
G = -2657.0646 a.u.
ΔG = 0 kJ/mol
ΔG = 1.05 kJ/mol
ΔG = 14.95 kJ/mol
ΔG = 19.50 kJ/mol
ΔG = 37.75 kJ/mol
ΔG = 38.01 kJ/mol
[B12I7]-
C-1
O-1
C-2
C-3
O-2
C-4
G = -2362.0607 a.u.
ΔG = 0 kJ/mol
ΔG = 17.18 kJ/mol
ΔG = 17.56 kJ/mol
ΔG = 26.28 kJ/mol
ΔG = 30.85 kJ/mol
ΔG = 37.39 kJ/mol
O-3
O-4
C-5
O-5
ΔG = 38.75 kJ/mol
ΔG = 40.15 kJ/mol
ΔG = 41.75 kJ/mol
ΔG = 44.33 kJ/mol
[B12I6]-
O-1
C-1
C-2
C-3
C-4
O-2
G = -2067.0747 a.u.
ΔG = 0 kJ/mol
ΔG = 2.38 kJ/mol
ΔG = 3.19 kJ/mol
ΔG = 15.51 kJ/mol
ΔG = 15.66 kJ/mol
ΔG = 17.73 kJ/mol
O-3
ΔG = 20.66 kJ/mol
[B12I5]-
O-1
O-2
O-3
O-4
O-5
G = -1772.1179 a.u.
ΔG = 0 kJ/mol
ΔG = 12.47 kJ/mol
ΔG = 19.90 kJ/mol
ΔG = 51.18 kJ/mol
ΔG = 69.39 kJ/mol
[B12I4]-
O-1
O-2
G = -1477.1256 a.u.
ΔG = 0 kJ/mol
ΔG = 22.55 kJ/mol
[B12I3]-
O-1
P-1
P-2
P-3
P-4
G = -1182.1618 a.u.
ΔG = 0 kJ/mol
ΔG = 14.32 kJ/mol
ΔG = 57.37 kJ/mol
ΔG = 57.93 kJ/mol
ΔG = 68.87 kJ/mol
[B12I2]-
P-1
P-2
P-3
G = -887.1885 a.u.
ΔG = 0 kJ/mol
ΔG = 26.31 kJ/mol
ΔG = 35.44 kJ/mol
[B12I1]-
P-1
P-2
P-3
G = -592.2248 a.u.
ΔG = 0 kJ/mol
ΔG = 31.42 kJ/mol
ΔG = 62.46 kJ/mol

Figure S4 The structures of the low-lying isomers of $[B_{12}I_n]^-$ (n = 11 – 1) optimized at the PBE0-D3(BJ)/aug-cc-pVTZ(-pp) level. The Gibbs free energies (G) of the global minimum structure and relative Gibbs free energies (ΔG) of each isomer calculated at the DLPNO-CCSD(T)/aug-cc-pVTZ(-pp) level with T = 20 K.

Table S3. (a) Methods and parameters for the CCS value calculation with IMoS. (b) EHSS/DHSS calculated CCS (Å^2) for all $[B_{12}I_n]^-$ (n = 11–1) isomers, along with values after linear scaling to match experimental trends.

**a** Method/parameter	
Used method	EHSS/DHSS
Number of orientations	3
Gas molecules per orientation	300000
Gas	N_2
Reduction coefficient	1.000
Molecular mass of Gas	28.00 Da
Alpha polarization	1.70 Å^3
Radius of gas	1.50 Å
Temperature	303 K
Pressure	313 Pa

**b** Structures	CCS (Å^2)	
	EHSS/DHSS	y = 1.337x − 1.3363
C-1$_{(11)}$	154.509	205.2422
C-1$_{(10)}$	150.6631	200.1003
C-2$_{(10)}$	151.5606	201.3002
C-1$_{(9)}$	144.5942	191.9861
C-2$_{(9)}$	146.217	194.1558
C-1$_{(8)}$	140.7533	186.8509
C-2$_{(8)}$	138.317	183.5935
C-3$_{(8)}$	139.7343	185.4885
C-1$_{(7)}$	133.5118	177.169
O-1$_{(7)}$	143.2458	190.1833
C-2$_{(7)}$	135.1136	179.3106
C-3$_{(7)}$	136.5977	181.2948
O-2$_{(7)}$	146.6952	194.7952
O-3$_{(7)}$	145.8306	193.6392
O-1$_{(6)}$	148.7211	197.5038
C-1$_{(6)}$	130.4209	173.0364

C-2(6)	129.02	171.1634
O-2(6)	146.0049	193.8723
O-3(6)	132.8942	176.3432
O-1(5)	135.9032	180.3663
O-2(5)	135.9587	180.4405
O-3(5)	141.7165	188.1387
O-1(4)	126.7665	168.1505
O-2(4)	126.508	167.8049
O-1(3)	115.0371	152.4683
		y = 1.4618x − 26.466
P-1(3)	129.193	171.3947
P-2(3)	123.206	163.3901
P-1(2)	118.4218	156.9936
P-2(2)	111.5155	147.7599
P-3(2)	111.3903	147.5925
P-11)	95.1748	125.9124
P-2(1)	82.478	108.9368

Theoretical calculations of collision cross sections (CCS) were performed using the EHSS/DHSS (Elastic/Diffuse Hard Sphere Scattering) method implemented in IMoS. This method reliably reproduces the relative trends in CCS but exhibits systematic deviations in absolute values. The main reason is that it oversimplifies ion–gas collisions as hard-sphere interactions, neglecting ion flexibility, charge distribution, and long-range interactions. Therefore, the absolute CCS values obtained from EHSS/DHSS should not be interpreted quantitatively.

To correct for the systematic offset between calculated and experimental CCS values, a linear scaling procedure was applied. For the cage-like and open structures, the relationship between the calculated CCS (x) and the scaled CCS (y) is given by:

$$y=1.337\,x-1.3363$$

For the planar structures, a separate linear scaling was employed, with the relationship between the calculated CCS (x) and the scaled CCS (y) given by:

$$y = 1.4618x - 26.466$$

This scaling preserves the relative trends while adjusting the absolute scale to better match experimental observations. It should be noted that the experimental absolute CCS values are also subject to uncertainty, as no dedicated calibration standard exists for this class of cluster ions (commonly used polyalanine calibrations are not fully applicable to this system). Therefore, both theoretical and experimental CCS values are best interpreted in a relative sense, which further justifies the use of the linear scaling procedure.

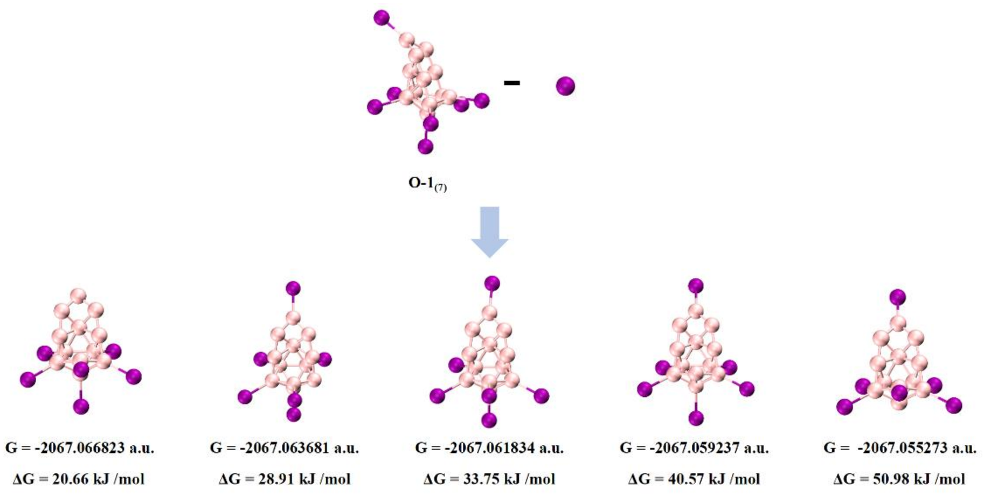

Figure S5 Starting from O-1$_{(7)}$, a series of $[B_{12}I_6]^-$ isomers were generated by removing one iodine atom, with ΔG values (T = 20 K) referenced to the lowest-energy $[B_{12}I_6]^-$ structure, O-1$_{(6)}$.

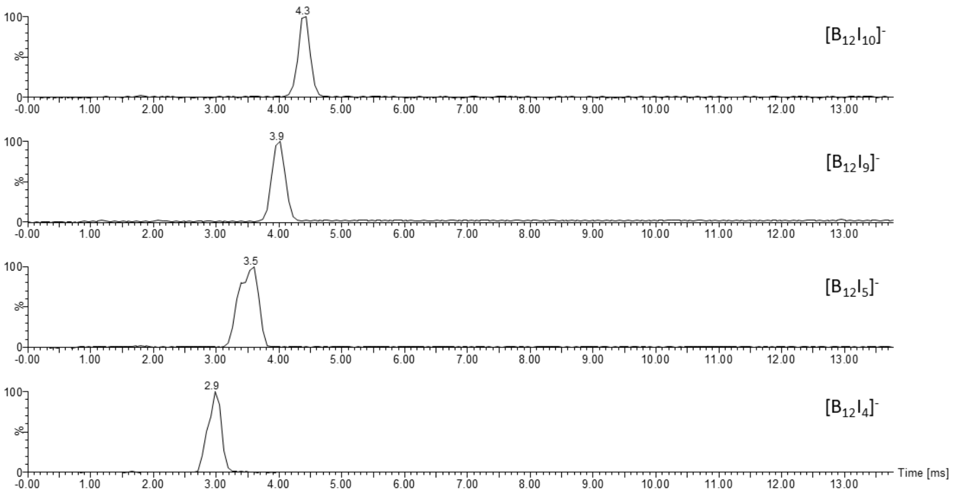

Figure S6 Comparison of $[B_{12}I_n]^-$ IMS data of n = 5 and 4 versus n = 10 and 9. The signal for n = 5 and 4 are noticeably broader indicating that several isomers with similar CCS are present.

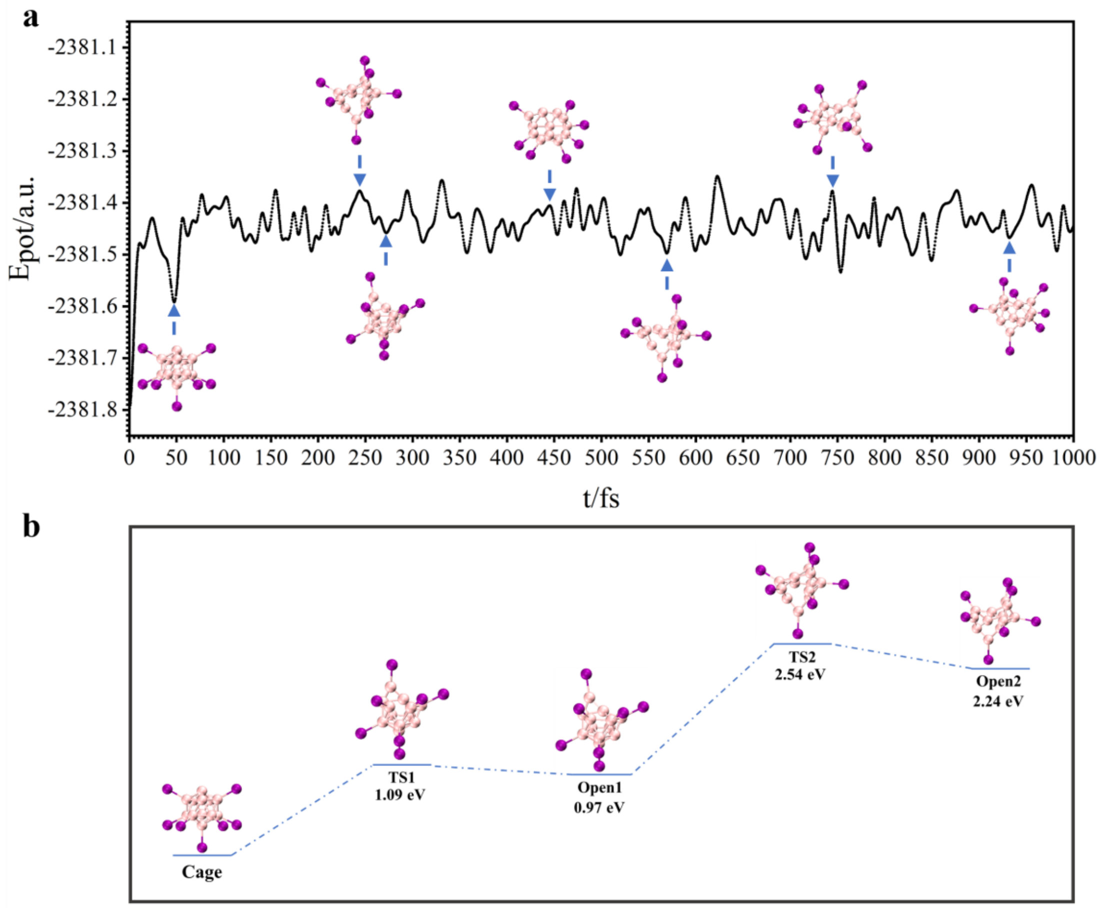

Figure S7. (a) The potential energy curve evolution stared with the global minimum of $[B_{12}I_7]^-$ C-1$_{(7)}$ upon being unfolded over time during ab initio molecular dynamics (AIMD) simulations conducted at the BHandHLYP-D3(BJ)/ma-def2-SVP level. (b) The transition-states (TSs) connecting different isomers of $[B_{12}I_7]^-$ were identified using the intrinsic reaction coordinate (IRC) method at the PBE0-D3(BJ)/def2-SVP level. The single-point (SP) energies were subsequently refined at the DLPNO-CCSD(T)/aug-cc-pVTZ(-pp) level.

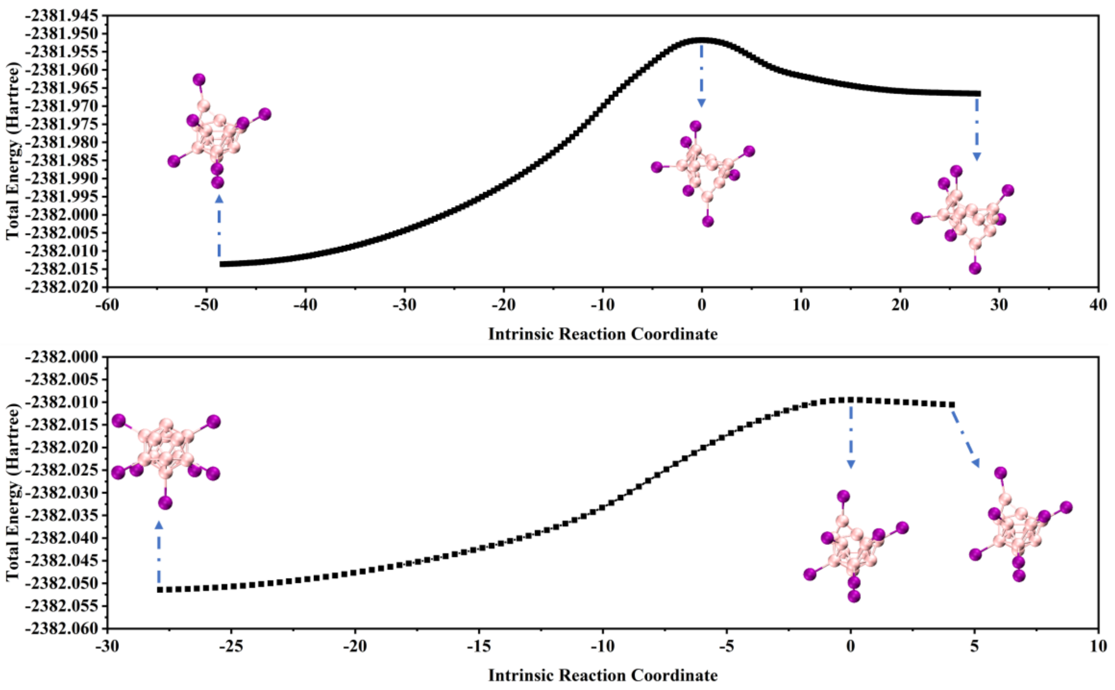

Figure S8 The IRC curves for the transition-states isomerization of $[B_{12}I_7]^-$ calculated at the PBE0-D3(BJ)/def2-SVP level.

To further confirm the identified $[B_{12}I_7]^-$ as critical turning points in the stepwise dissociation process transforming boron core topology from 3D to 2D, ab initio molecular dynamics (AIMD) simulations were employed to reveal their dynamical stabilities. The structural evolution of the fragmentation cluster of $[B_{12}I_7]^-$ is reproduced by AIMD simulation as shown in **Figure S7**. The cage-like $[B_{12}I_7]^-$ gradually evolves into an open structure, which is consistent with our previous observation from quantum chemical calculations. Due to the large size of $[B_{12}I_7]^-$ cluster, more significant structural diversity has emerged in the AIMD simulations, confirming the 3D-to-2D structural transitions during the dissociation process. Furthermore, the intrinsic reaction coordinate (IRC) calculations based on the transition state theory were performed to investigate the involving interconversion process between 3D and quasi-3D/2D isomers (**Figure S8**). The possible reaction pathways involving the transition states transitioning from a cage-like structure to an open or planar structure were successfully identified for $[B_{12}I_7]^-$ (**Figure S7b**). For instance, the $[B_{12}I_7]^-$ cluster firstly overcomes an energy barrier of 1.09 eV to convert to the intermediate open1 form, and subsequently a barrier of 1.57 eV to achieve the open2

form. It should be noted that the fragmentation clusters in current experiments were generated through collisions, providing sufficiently high kinetic energy to overcome the existing conversion barriers. Once the collisions and cross-the-barrier are completed, with the following subsequent cluster system cooling down, these formed low-lying isomers become difficult to reconvert, thereby kinetically trapped in their respective forms.

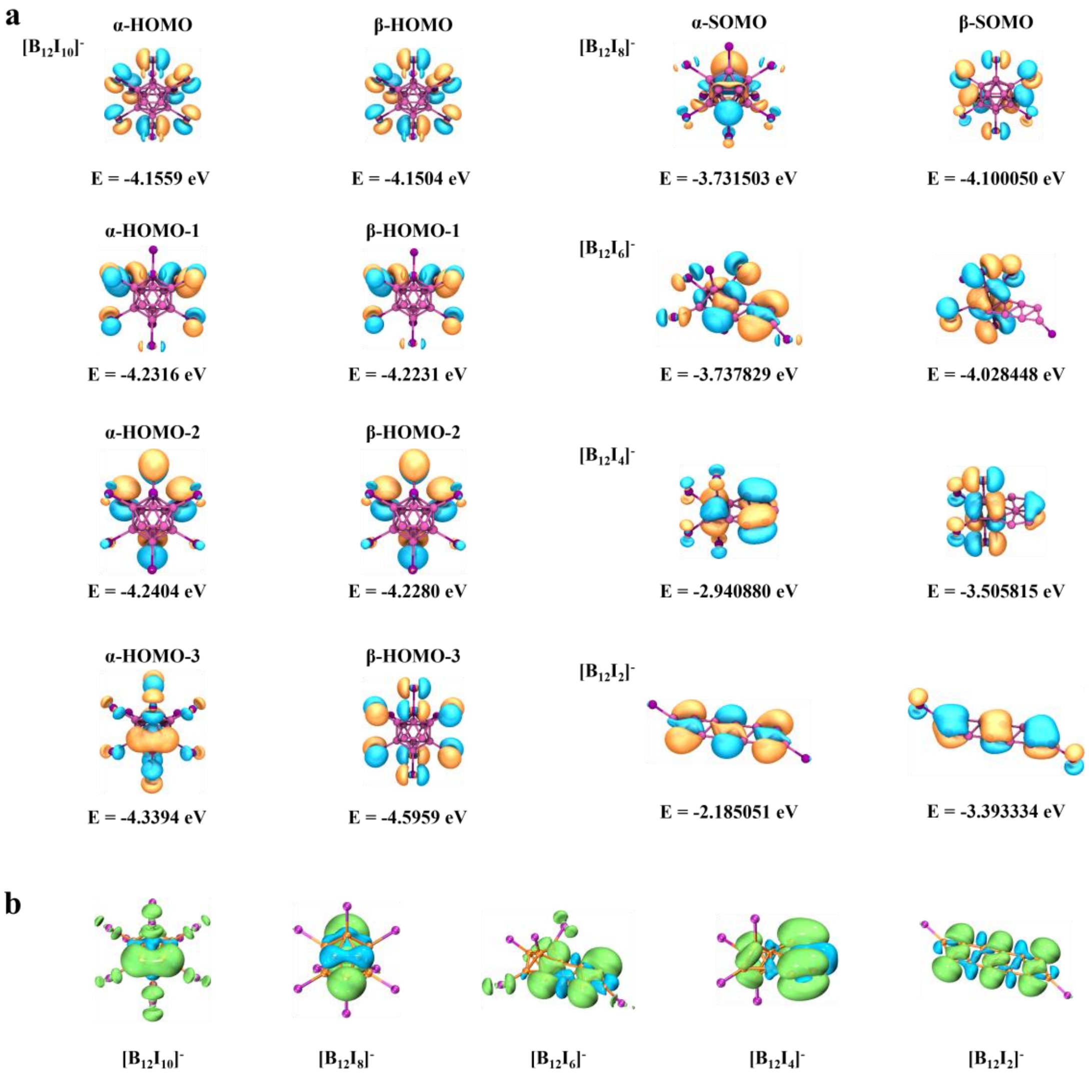

Figure S9 (a) SOMOs (isovalue = 0.03) and (b) spin density (isovalue = 0.001) of the open-shell $[B_{12}I_n]^-$ (even n).

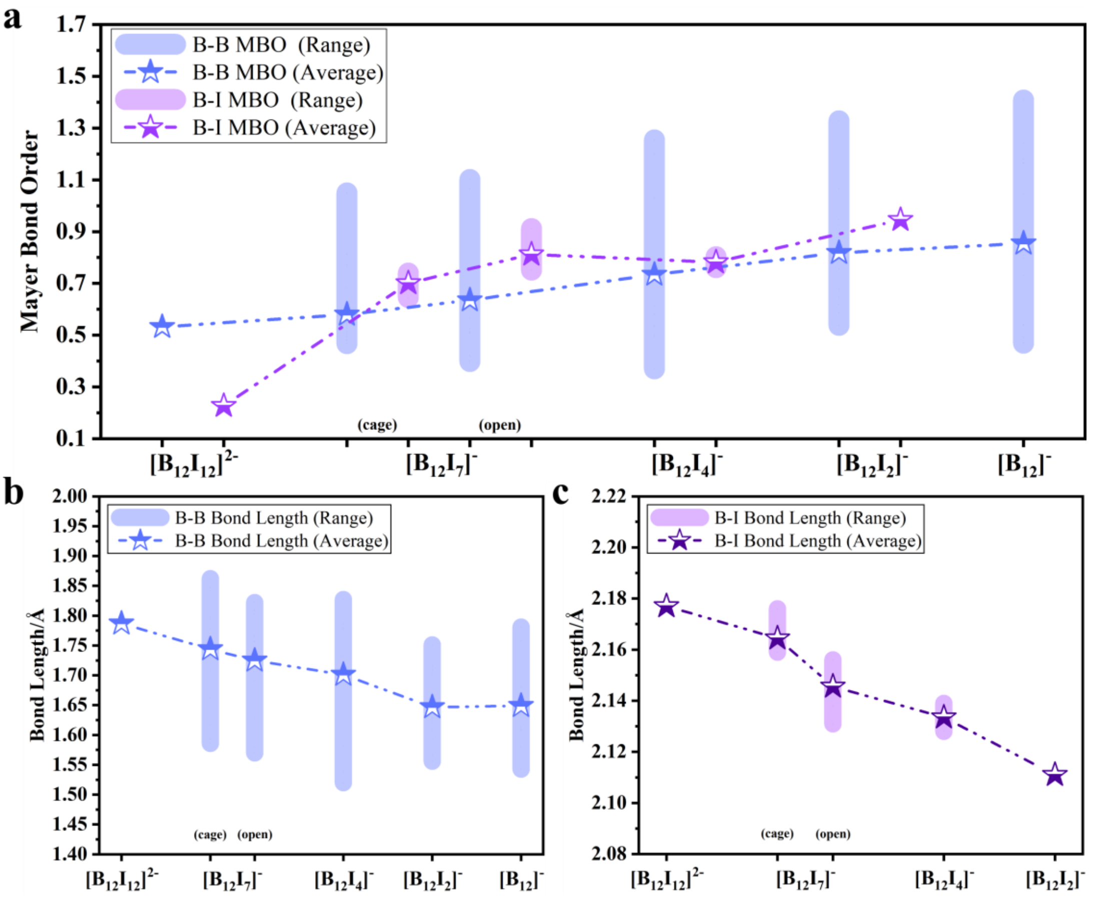

Figure S10. Calculated Mayer bond orders (MBO) of B-B and B-I bonds for selected $[B_{12}I_n]^-$ clusters at the PBE0/cc-pVTZ(-pp) level with the averaged MBO values and MBO ranges indicated (a). Calculated bond lengths of B-B bonds (b) and B-I bonds (c) for selected $[B_{12}I_n]^-$ clusters with both average bond lengths and ranges indicated.

cage

1c-2e

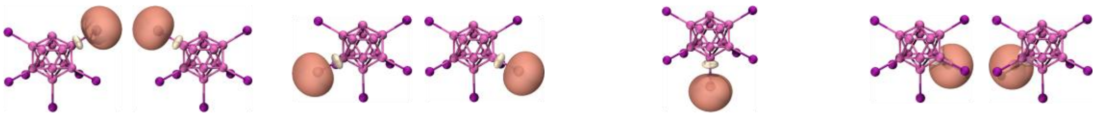

ON=1.99 | e | ON=1.98 | e | ON=1.98 | e | ON=1.98 | e |

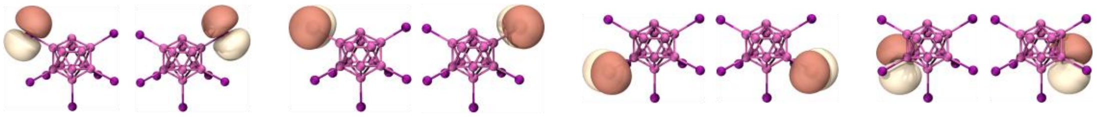

ON=1.94 | e | ON=1.94 | e | ON=1.93 | e | ON=1.93 | e |

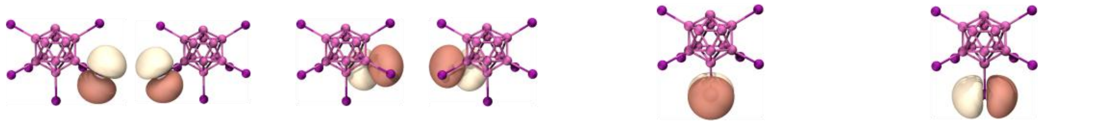

ON=1.93 | e | ON=1.93 | e | ON=1.93 | e | ON=1.93 | e |

2c-2e

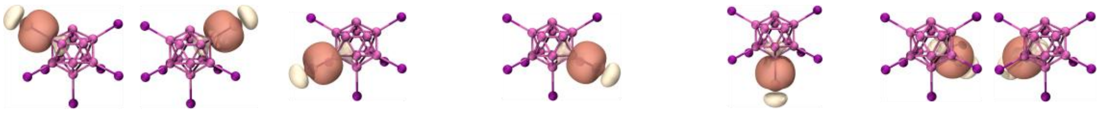

ON=1.98 | e | ON=1.98 | e | ON=1.98 | e | ON=1.97 | e | ON=1.96 | e |

3c-2e

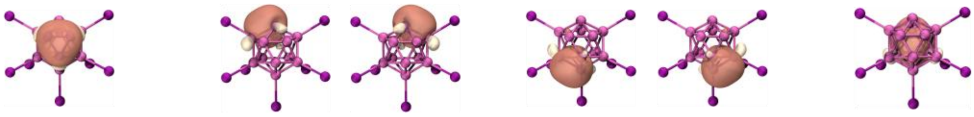

ON=1.90 | e | ON=1.88 | e | ON=1.86 | e | ON=1.85 | e |

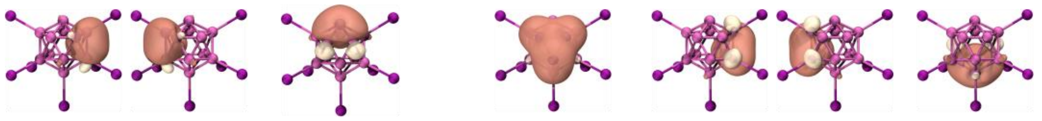

ON=1.85 | e | ON=1.79 | e | ON=1.78 | e | ON=1.77 | e | ON=1.74 | e |

12c-2e

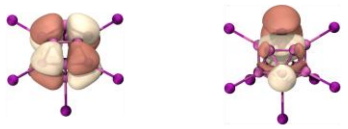

ON=2.00 | e | ON=1.96 | e |

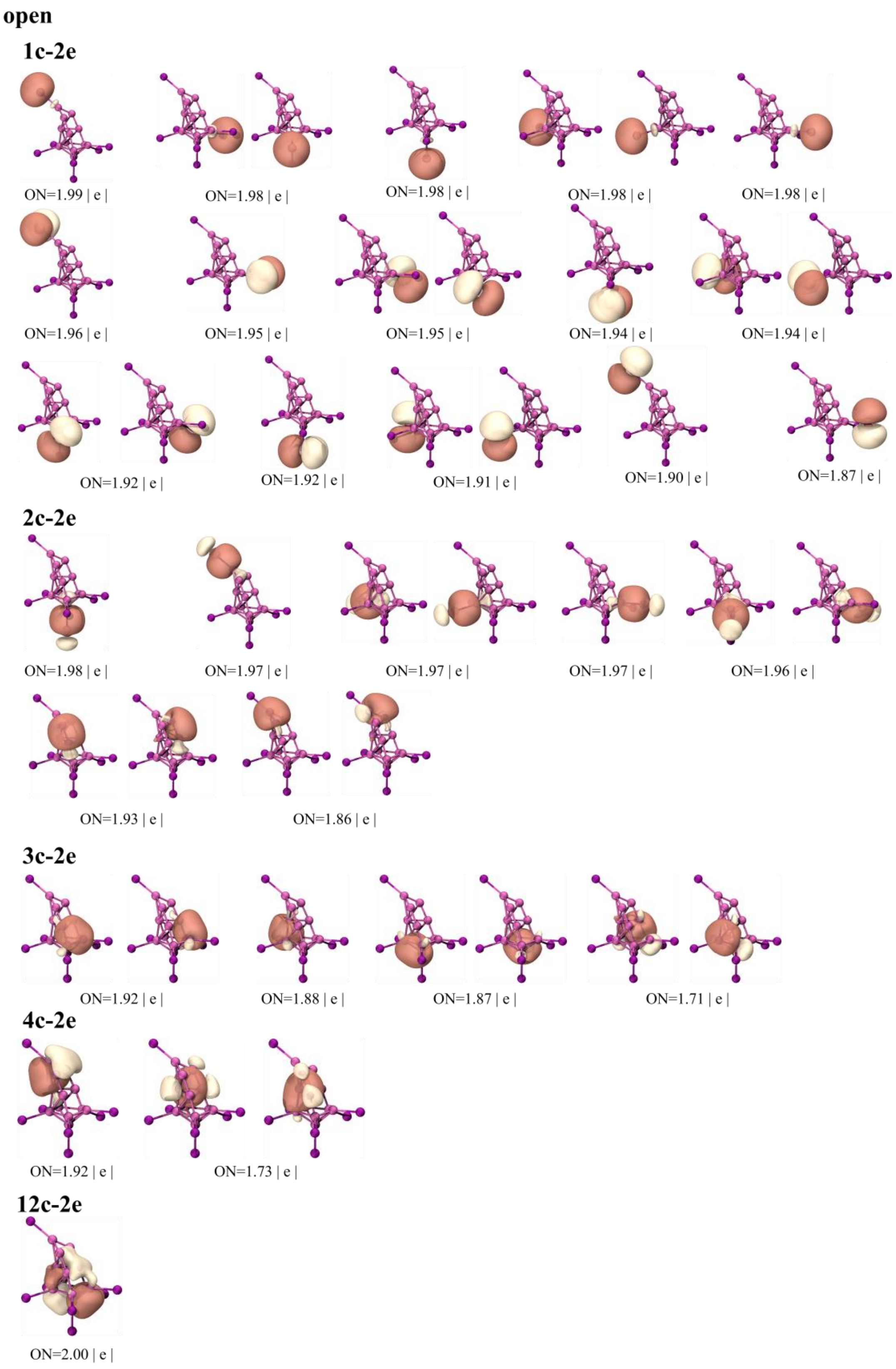

Figure S11 The adaptive natural density partitioning (AdNDP) analysis for the coexisted isomers of $[B_{12}I_7]^-$ at PBE0-D3(BJ)/aug-cc-pVTZ(-pp) level with an isovalue of 0.03. Occupation number (ON) is equal to 2.00 |e| in the ideal case.

The adaptive natural density partitioning (AdNDP) method as an ideal tool for better deciphering the nature of bond localization and delocalization were performed for both cage and open structures of $[B_{12}I_7]^-$. The AdNDP method can represent the electronic structure in terms of n-center two-electron (nc–2e) bonds, where n ranges from 1 to the maximum number of atomic centers. AdNDP analysis for $[B_{12}I_7]^-$ revealed 7 plus 14 1c–2e lone pairs (LPs) of electrons in the p and d orbitals of I ligands. The cage structure identifies 7 localized B−I σ bonds at the edge, 13 delocalized 3c–2e bonds cross the boron cage, and 2 delocalized 12c–2e π and σ bonds. In contrast, the open structure possesses 7 localized B−I σ bonds at the edge and 4 localized B−B σ bonds, 7 delocalized 3c–2e σ bonds, 3 delocalized 4c–2e σ bonds, and 1 delocalized 12c–2e hybrid π/σ bonds with significant deformation, suggesting more diverse bonds formation due to the distortion of cage structure. More importantly, this π plus σ electron delocalization contributes to the stability of open structure of $[B_{12}I_7]^-$.

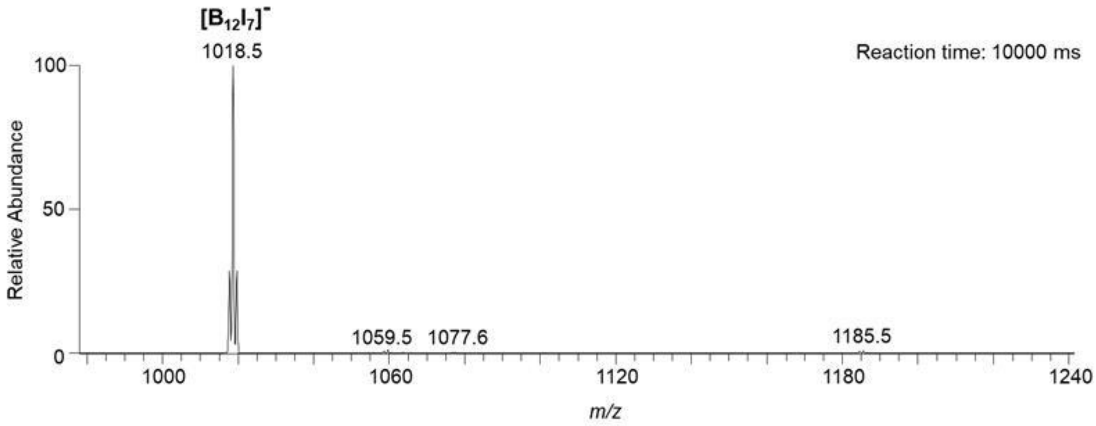

Figure S12. Mass spectrum obtained for isolation of $[B_{12}I_7]^-$ and a reaction time of ten seconds with allyl iodide introduced to the ion trap.

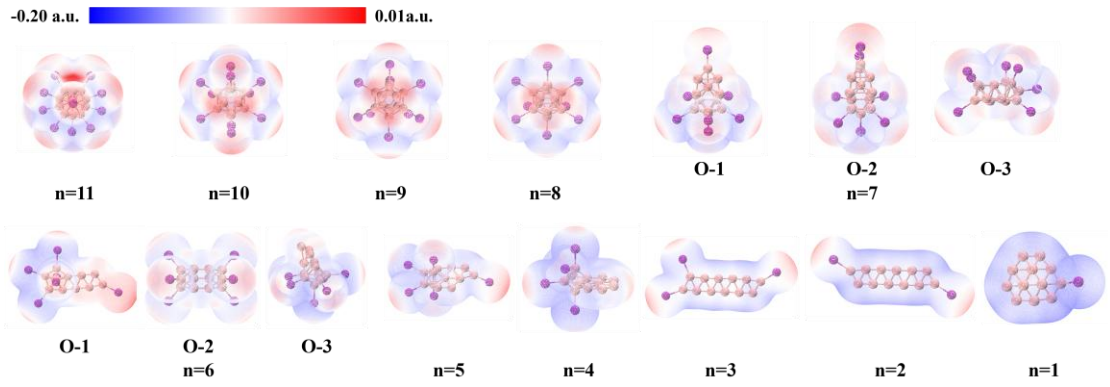

Figure S13. Electrostatic potential (ESP) plotted on the molecular surface (isovalue = 0.001) of $[B_{12}I_n]^-$ ($n = 11 – 1$).

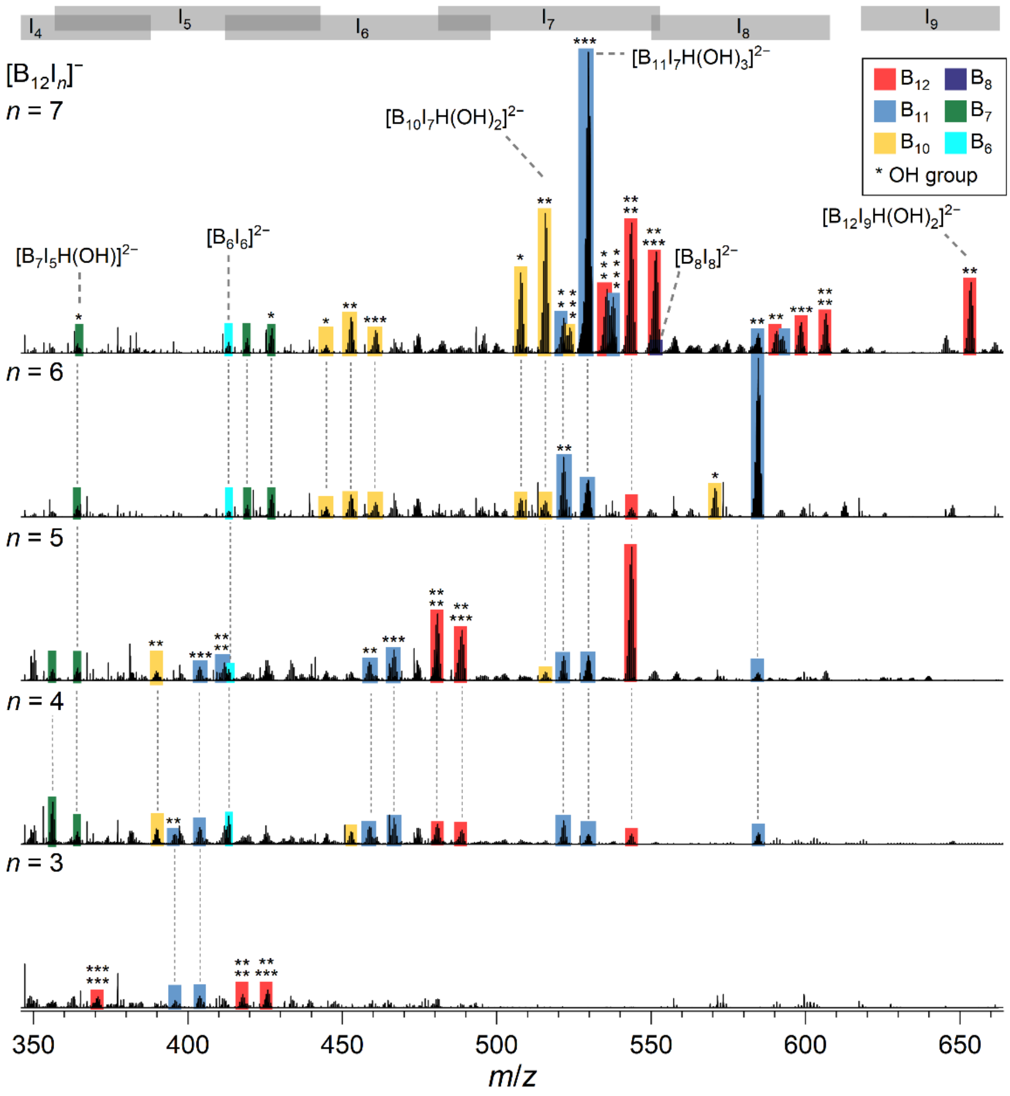

Figure S14. (-)ESI mass spectra of anions extracted from a layer formed by deposition of 0.1 nmol $[B_{12}I_n]^-$ (n = 7 to 3, top to bottom), using acetonitrile as extraction solvent. Note that scaling of the y-axis is fixed for all spectra. Background ions from the solvent and surface contamination were subtracted; further details on the background subtraction procedure are provided in **Figure S15**. A color code labels for abundant ions of different boron cluster sizes. The *m/z* ranges corresponding to particular numbers of iodine substituents are marked at the top. Numbers of OH-substituents are indicated by *.

Ions were deposited using ion kinetic energies of approximately 7-10 eV (maximum of the kinetic energy probability function). ESI-MS analysie of the deposited layers revealed for all deposited $[B_{12}I_n]^-$ anions, complex product mixtures.

Deposition of $[B_{12}I_n]^-$ ions with $n < 7$ leads to the formation of smaller *closo*-borate anions on the surface. The mass spectrum (**Figure S14**, top) shows $[B_mX_m]^{2-}$ ions with $m = 12$, 11, 10, 8, 7 and 6. The substituents (X) found are I, OH, H, the latter likely originating from adsorbed water at the interface or background gases of the vacuum chamber. Remarkably, no $[B_9X_9]^{2-}$ ions were found. Ions with surprisingly high iodine content were observed in all cases. Apparently, the selected singly charged ions become doubly charged either by redox reactions at the conductive surface or by binding an anion like I^-. Such products with a greater number of iodine atoms in the substituent shell than present in the mass-selected fragment demonstrate that iodine atoms or iodide anions may be bound at the surface. Such iodine may originate from other deposited anions, which are decomposed at the surface upon collisions, and is available for recombination with vacant boron sites of other ions. The lower stability of $[B_{12}I_6]^-$ compared to $[B_{12}I_7]^-$ and $[B_{12}I_5]^-$ (based on CID and NIPES experiments) is also reflected in the product distribution found on the surface: despite similar kinetic energies of the deposited ions, more B_{12}-unit degradation and a larger amount of available iodine at the interface was found for $[B_{12}I_6]^-$. Although similar numbers of ions were deposited, absolute amounts of the detected products diminish with smaller n. Apparently, stabilizing reactions forming dianionic *closo*-borate ions is less probable for lower substitution levels – in particular for (quasi-)planar ions – and degradation into volatile products becomes a dominant pathway. However, for open $[B_{12}I_n]^-$ (n = 7–4), preparative mass spectrometry may open new pathways to generate conjugated structures of small *closo*-borate anions with reagents available at the interface.

Table S4 Products anions identified by HR-MS analysis of the dissolved layer after fragment ion deposition of $[B_{12}I_n]^-$ (n = 3-7). Presence of a certain anion in the respective deposition experiment is indicated by a green checkmark. The three most intense signals per n are marked in decreasingly saturated orange color. The annotated *m*/*z* values correspond to the highest abundance signal in the isotopic pattern of the assigned ion.

m/*z*	Assignment	$[B_{12}I_3]^-$	$[B_{12}I_4]^-$	$[B_{12}I_5]^-$	$[B_{12}I_6]^-$	$[B_{12}I_7]^-$
356.3	$[B_7I_5H_2]^{2-}$		✓			
364.3	$[B_7I_5H(OH)]^{2-}$		✓	✓		
370.9	$[B_{12}I_4H_2(OH)_6]^{2-}$	✓				
381.9	$[B_{10}I_5H_4(OH)]^{2-}$		✓	✓		
389.8	$[B_{10}I_5H_3(OH)_2]^{2-}$		✓	✓		
395.8	$[B_{11}I_5H_4(OH)_2]^{2-}$		✓			
397.8	$[B_{10}I_5H_2(OH)_3]^{2-}$		✓	✓		
403.8	$[B_{11}I_5H_3(OH)_3]^{2-}$	✓	✓	✓		
411.8	$[B_{11}I_5H_2(OH)_4]^{2-}$	✓	✓	✓		
413.2	$[B_6I_6]^{2-}$		✓			✓
417.8	$[B_{12}I_5H_3(OH)_4]^{2-}$	✓	✓			
419.3	$[B_7I_6H]^{2-}$	✓	✓	✓	✓	✓
425.3	$[B_8I_6H_2]^{2-}$		✓			
425.8	$[B_{12}I_5H_2(OH)_5]^{2-}$	✓	✓	✓		
427.2	$[B_7I_6(OH)]^{2-}$				✓	✓
431.3	$[B_9I_6H_3]^{2-}$		✓			
433.8	$[B_{12}I_5H(OH)_6]^{2-}$	✓	✓	✓		
436.8	$[B_{10}I_6H_4]^{2-}$		✓	✓		
444.8	$[B_{10}I_6H_3(OH)]^{2-}$		✓	✓	✓	✓
452.8	$[B_{10}I_6H_2(OH)_2]^{2-}$		✓		✓	✓
458.8	$[B_{11}I_6H_3(OH)_2]^{2-}$		✓	✓		
460.8	$[B_{10}I_6H(OH)_3]^{2-}$		✓		✓	✓
466.8	$[B_{11}I_6H_2(OH)_3]^{2-}$		✓	✓	✓	✓
474.8	$[B_{11}I_6H(OH)_4]^{2-}$		✓	✓	✓	✓
480.8	$[B_{12}I_6H_2(OH)_4]^{2-}$		✓	✓		
482.2	$[B_7I_7]^{2-}$					✓
488.8	$[B_{12}I_6H(OH)_5]^{2-}$		✓	✓		
496.2	$[B_8I_7(OH)]^{2-}$					✓
499.7	$[B_{10}I_7H_3]^{2-}$					✓
507.7	$[B_{10}I_7H_2(OH)]^{2-}$		✓		✓	✓
515.7	$[B_{10}I_7H(OH)_2]^{2-}$		✓		✓	✓
521.7	$[B_{11}I_7H_2(OH)_2]^{2-}$		✓	✓	✓	✓
523.7	$[B_{10}I_7(OH)_3]^{2-}$					✓
529.7	$[B_{11}I_7H(OH)_3]^{2-}$		✓	✓	✓	✓
535.7	$[B_{12}I_7H_2(OH)_3]^{2-}$					✓

537.7	$[B_{11}I_7(OH)_4]^{2-}$		✓			✓
543.7	$[B_{12}I_7H(OH)_4]^{2-}$		✓	✓	✓	✓
551.7	$[B_{12}I_7(OH)_5]^{2-}$		✓	✓		✓
562.7	$[B_{10}I_8H_2]^{2-}$				✓	
565.2	$[B_9I_8(OH)]^{2-}$					✓
570.7	$[B_{10}I_8H(OH)]^{2-}$				✓	✓
578.7	$[B_{10}I_8(OH)_2]^{2-}$					✓
584.7	$[B_{11}I_8H(OH)_2]^{2-}$		✓	✓	✓	✓
590.7	$[B_{12}I_8H_2(OH)_2]^{2-}$	✓	✓			✓
592.7	$[B_{11}I_8(OH)_3]^{2-}$				✓	✓
598.7	$[B_{12}I_8H(OH)_3]^{2-}$					✓
602.7	$[B_7I_4H_2(NH_2)]^{2-}[H]^+$		✓			
606.7	$[B_{12}I_8(OH)_4]^{2-}$			✓	✓	✓
618.7	$[B_7I_4H(OH)(NH_2)]^{2-}[H]^+$		✓			
625.6	$[B_{10}I_9H]^{2-}$				✓	
640.0	$[B_{24}I_{12}H_9OH_8]^{3-}$			✓		
645.6	$[B_{12}I_9H_2(OH)]^{2-}$					✓
647.6	$[B_{11}I_9(OH)_2]^{2-}$		✓		✓	
653.6	$[B_{12}I_9H(OH)_2]^{2-}$					✓
661.6	$[B_{12}I_9(OH)_3]^{2-}$					✓

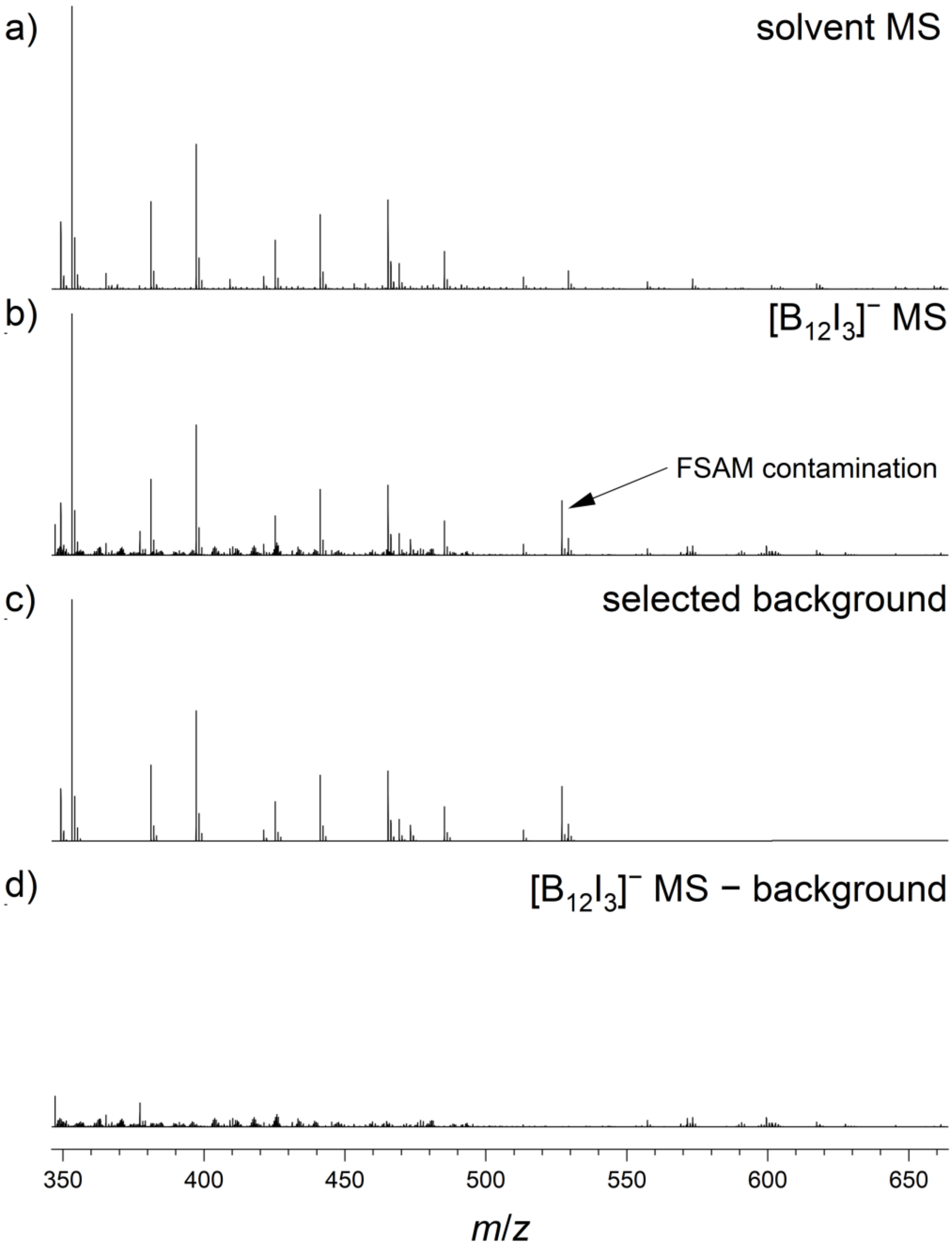

Figure S15. Example of the background removal procedure applied to the MS spectra acquired after dissolution of the surface layers generated by fragment ion deposition of $[B_{12}I_n]^-$ (here: n=3). (a) (-)ESI-MS spectrum of the pure solvent used for dissolving the layer (acetonitrile). (b) (-)ESI-MS spectrum of the solution resulting from dissolving the layer generated by $[B_{12}I_3]^-$ deposition in acetonitrile. Note that the marked signal stems from contamination due to the FSAM-covered surface used as deposition substrate and was therefore also selected for removal. (c) Selected background from the solvent background (a) and FSAM contamination (b) that was used to subtract from the sample MS. (d) Resulting spectrum after background subtraction showing only signals due to species in the deposited layer.

Table S5. Settings/conditions applied during fragment ion deposition.

**Parameter**	**$[B_{12}I_7]^-$**	**$[B_{12}I_6]^-$**	**$[B_{12}I_5]^-$**	**$[B_{12}I_4]^-$**	**$[B_{12}I_3]^-$**
**Precursor**	$K_2[B_{12}I_{12}]$	$K_2[B_{12}I_{12}]$	$K_2[B_{12}I_{12}]$	$K_2[B_{12}I_{12}]$	$K_2[B_{12}I_{12}]$
**c (solution) / mM**	0.1	0.1	0.1	0.1	0.1
**flow rate / mL/h**	0.12	0.12	0.12	0.12	0.12
**U (ESI) / V**	-3000	-3000	-3400	-3100	-3300
**T (Inlet 1, 2) / °C**	120	120	120	120	120
**U (Inlet 1) / V**	-400	-400	-450	-420	-400
**U (Inlet 2) / V**	-400	-400	-450	-420	-400
**f (HPF) / kHz**	676	676	676	676	676
**U (HPF 1) / V**	-450	-450	-450	-420	-430
**U (HPF 2) / V**	-320	-260	-300	-300	-290
**U (HPF 3) / V**	-330	-290	-330	-330	-293
**U (HPF 4) / V**	-210	-160	-200	-200	-195
**p (HPF) / Torr**	7.3	7.2	7.3	7.3	7.3
**f (LPF) / kHz**	881	881	881	881	881
**U (LPF 1) / V**	-225	-160	-195	-195	-221
**U (LPF lens) / V**	-190	-180	-160	-160	-178
**U (LPF 2) / V**	-108	-110	-148	-154	-154
**p (LPF) / Torr**	2.3	2.3	2.3	2.3	1.5
**f (CC) / kHz**	1778	1778	1778	1778	1778
**U (CC bias) / V**	-4.8	-2.9	-0.8	-2.8	-3.0
**U (CC lens) / V**	-11.3	-8.5	-12.3	-12.3	-19.4
**f (BIG) / kHz**	1773	1773	1773	1773	1773
**U (BIG bias) / V**	-6.7	-4.5	-5.3	-6.3	-1.3
**U (BIG lens) / V**	40	65	33	83	25
**f (QMF) / kHz**	550	550	550	550	550
**U (QMF in) / V**	120	50	90	80	110
**U (QMF pre) / V**	-3	50	-4	-4	22
**U (QMF post) / V**	2	30	23	63	7
**U (QMF out) / V**	10	8	10	20	8
**U (QMF bias) / V**	60	102	53	34	42
m*/*z	1020.0	880.0	770.0	637.0	512.0
**Δ *m*/*z***	45.0	45.0	40.0	50.0	50.0
**U (L1) / V**	300	300	300	300	300
**U (L2) / V**	100	100	100	100	100
**U (L3) / V**	300	300	300	300	300
**p (SL chamber) / mbar**	$5\text{-}7x10^{-5}$	$5\text{-}7x10^{-5}$	$5\text{-}7x10^{-5}$	$5\text{-}7x10^{-5}$	$5\text{-}7x10^{-5}$
**KE / eV**	8.0	7.0	8.0	7.5	9.7

Additional experimental details on fragment ion deposition

The two electrospray ionization (ESI) sources of a previously described ion soft-landing instrument optimized for fragment ion deposition[2] were used to transfer the ions from solution into the gas phase. 0.1 mM solutions of $K_2[B_{12}I_{12}]$ in acetonitrile (ACN) were used as the precursor solution for the generation of fragment ions $[B_{12}I_n]^-$ (n = 7-3). Two ion funnels operating at high and low pressure (high-pressure ion funnel (HPF) and low-pressure ion funnel (LPF)), respectively, focused the ion beam and transferred the gaseous ions from the source region to a rough vacuum stage. Downstream of the ion funnels, CID of the precursor ions was carried out in a collision cell (CC) by applying a voltage difference to ion optics elements. Multiple collisions with background gas molecules lead to fragmentation reactions. The use of a 90° bent ion guide (BIG) enabled the separation of the ion beam from the stream of neutral molecules originating from the source. Ions were then selected by a quadrupole mass filter (QMF) based on their mass-to-charge ratio and guided to the deposition surface, as described previously. [2]In front of the deposition surfaces, a series of three lenses was mounted to focus the ion beam (L1; L2 and L3), as described previously.[3] The deposition surface consisted of a silicon wafer (1 × 1 cm^2) covered with a 30 nm thick gold adlayer that is bound to the underlying Si surface *via* a 5 nm thick chromium adhesion layer (Siegert Wafer GmbH, Aachen, Germany). Prior to soft-landing experiments, the surface was cleaned by successively immersing the sample in Millipore water and high purity ethanol under ultrasonication for at least three minutes. Subsequently, remaining contaminations were removed by combined UV/ozone cleaning (Ossila UV Ozone Cleaner L2002A, Ossila Limited, Sheffield, UK). The conductive gold surface was grounded *via* a picoammeter, which measured the current of soft-landed ions during the deposition. The number of deposited ions was derived by integrating the ion current over time. The ion optics are powered and controlled by the MIPS control system developed by GAA Custom Electronics, LLC and were adjusted to obtain a maximum ion current after mass selection. Applied RF voltages were adjusted to the resonance frequency of the electric LC circuit. Table S4 gives a list of settings used during ion deposition.

Measurement of the kinetic energy (KE) of the deposited fragment ions was performed with the retarding potential method, see previous detailed descriptions.[4-5] Below, we show the results for the ions as reported in Table S3. We state the most probable KE as determined from the maximum of the probability density curve that results from integrating the sigmoidal fit of the average of five individual measurements.

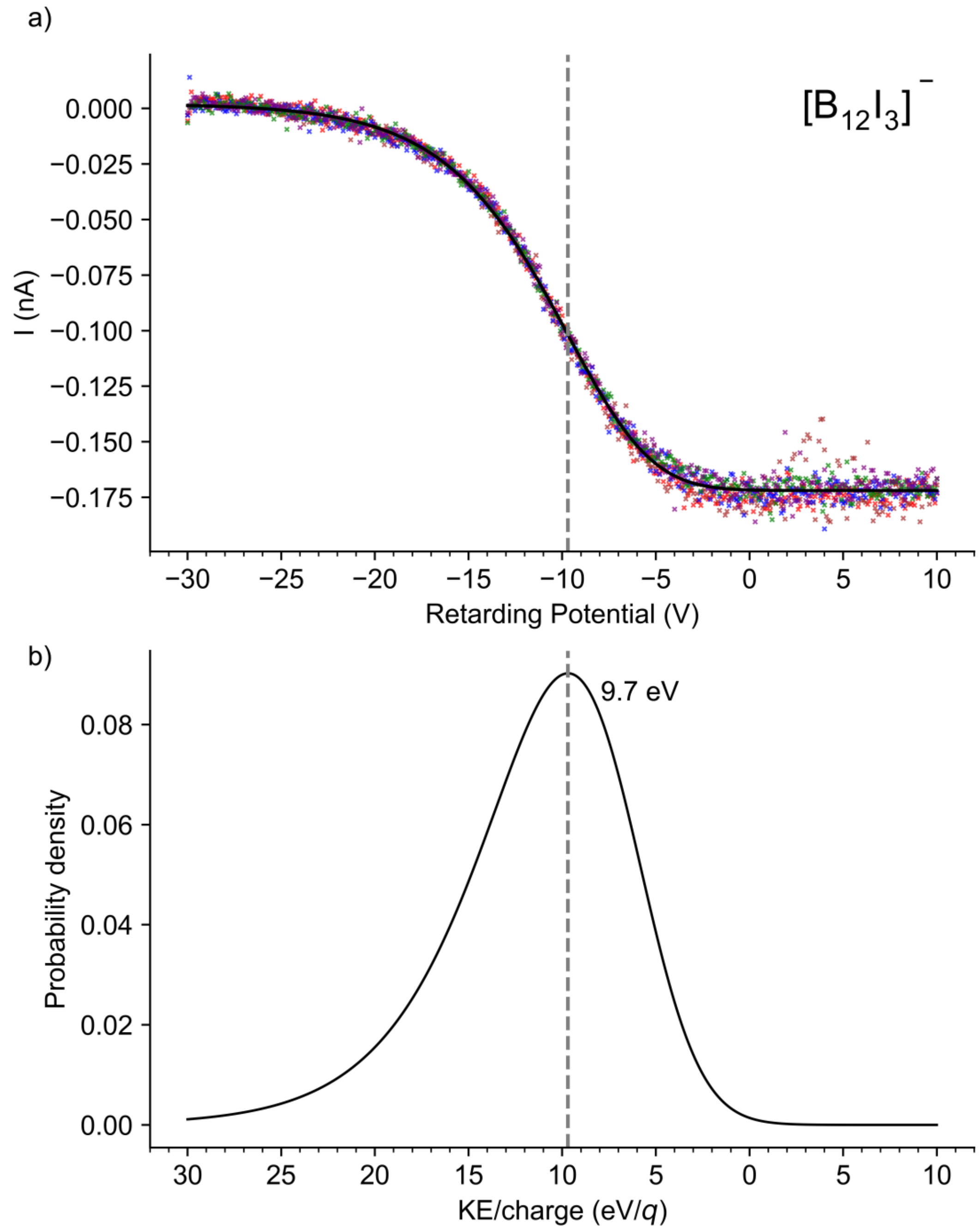

Figure S16. (a) Ion current of $[B_{12}I_3]^-$ as a function of the retarding potential. Five separate experiments under identical conditions were performed (individual data points are shown as "X" in a color representative for a single experiment) and all data was subsequently averaged and fitted by a sigmoidal function (black line). The position of the inflection point is marked with a dashed grey line. (b) Probability density function for the KE per charge (*q*) obtained by calculating the first derivative of the sigmoidal function in (a). The position of the maximum is marked with a dashed grey line and can be understood as most probable KE.

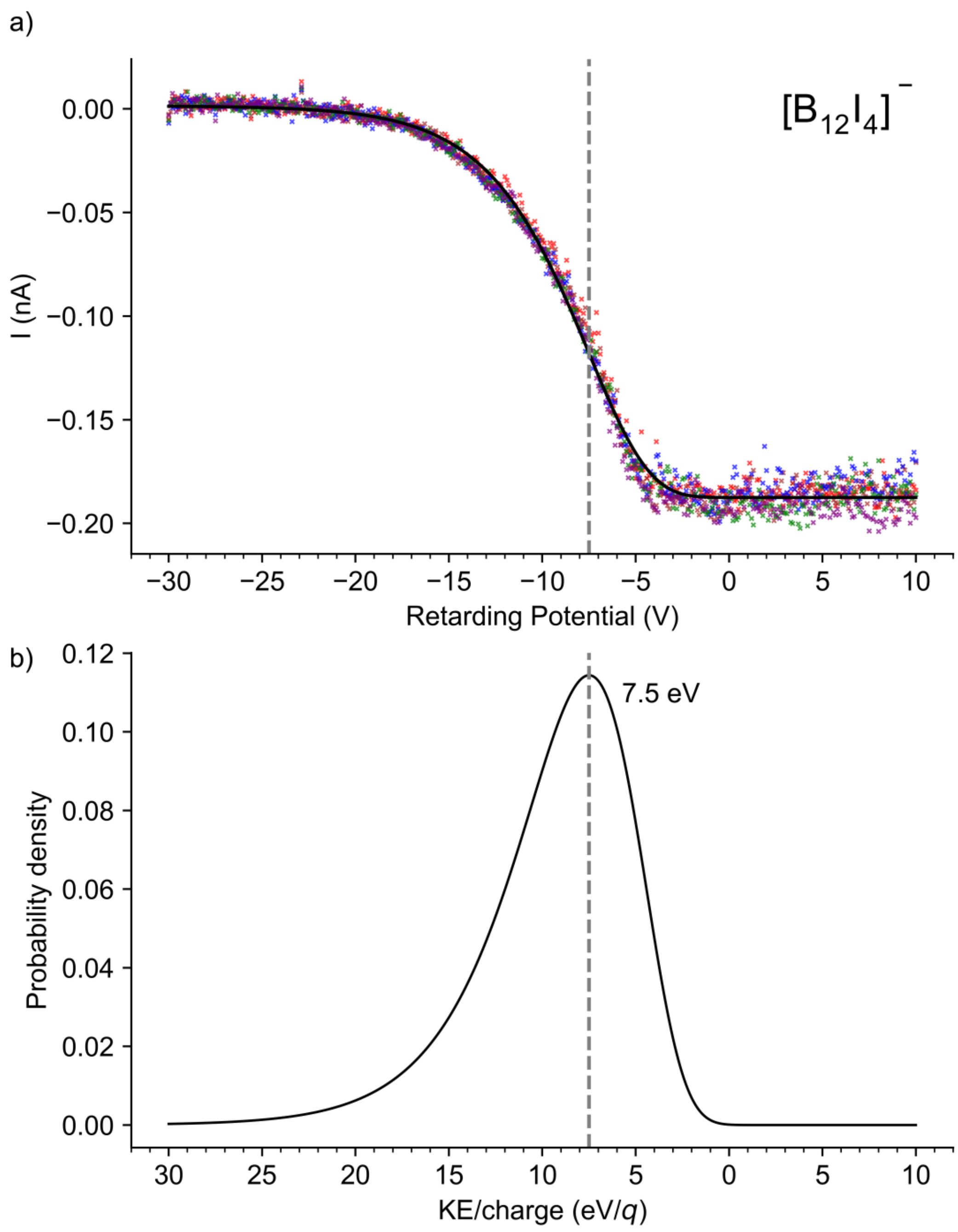

Figure S17. (a) Ion current of $[B_{12}I_4]^-$ as a function of the retarding potential. Five separate experiments under identical conditions were performed (individual data points are shown as "X" in a color representative for a single experiment) and all data was subsequently averaged and fitted by a sigmoidal function (black line). The position of the inflection point is marked with a dashed grey line. (b) Probability density function for the KE per charge (q) obtained by calculating the first derivative of the sigmoidal function in (a). The position of the maximum is marked with a dashed grey line and can be understood as most probable KE.

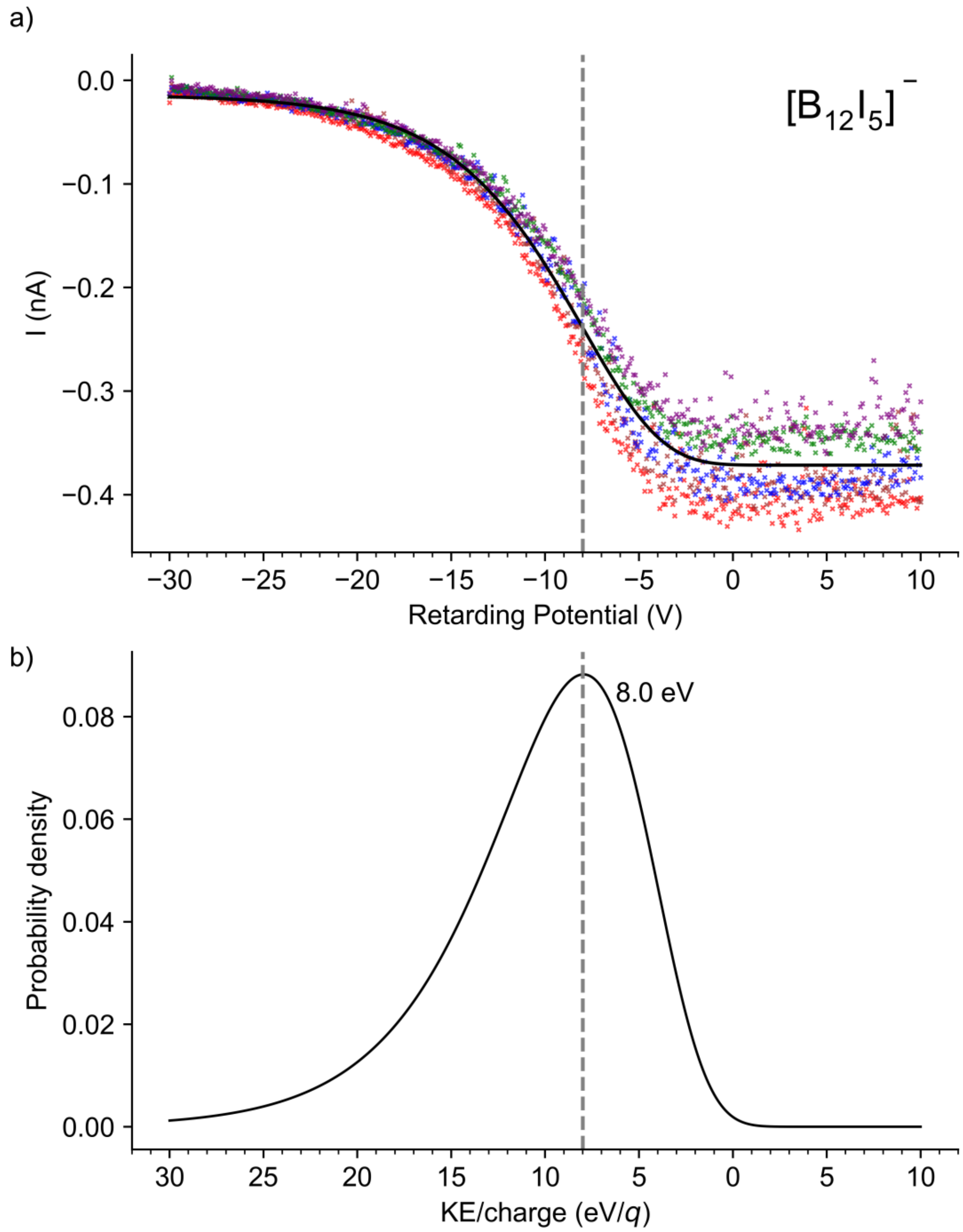

Figure S18. (a) Ion current of $[B_{12}I_5]^-$ as a function of the retarding potential. Five separate experiments under identical conditions were performed (individual data points are shown as "X" in a color representative for a single experiment) and all data was subsequently averaged and fitted by a sigmoidal function (black line). The position of the inflection point is marked with a dashed grey line. (b) Probability density function for the KE per charge (*q*) obtained by calculating the first derivative of the sigmoidal function in (a). The position of the maximum is marked with a dashed grey line and can be understood as most probable KE.

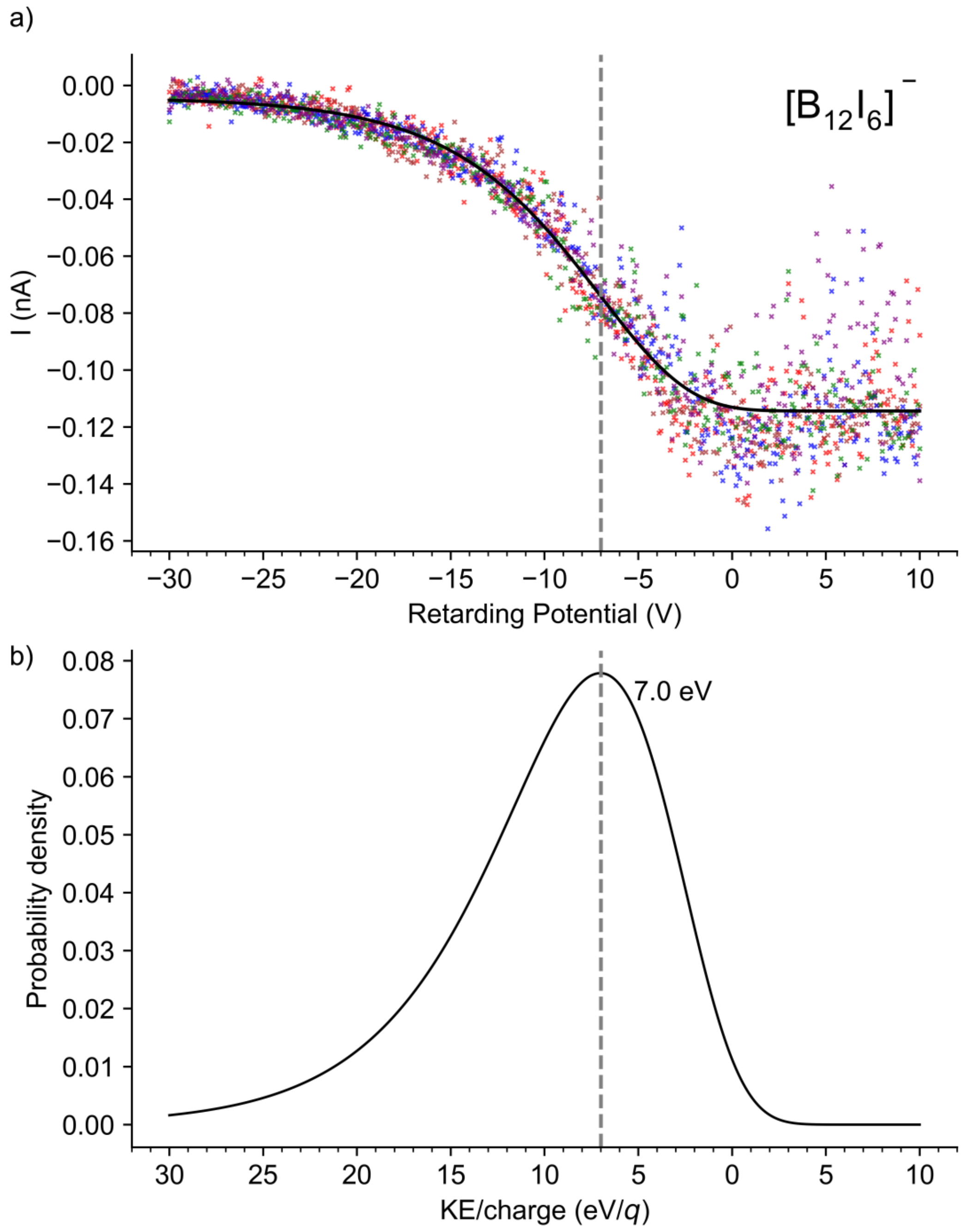

Figure S19. (a) Ion current of $[B_{12}I_6]^-$ as a function of the retarding potential. Five separate experiments under identical conditions were performed (individual data points are shown as "X" in a color representative for a single experiment) and all data was subsequently averaged and fitted by a sigmoidal function (black line). The position of the inflection point is marked with a dashed grey line. (b) Probability density function for the KE per charge (q) obtained by calculating the first derivative of the sigmoidal function in (a). The position of the maximum is marked with a dashed grey line and can be understood as most probable KE.

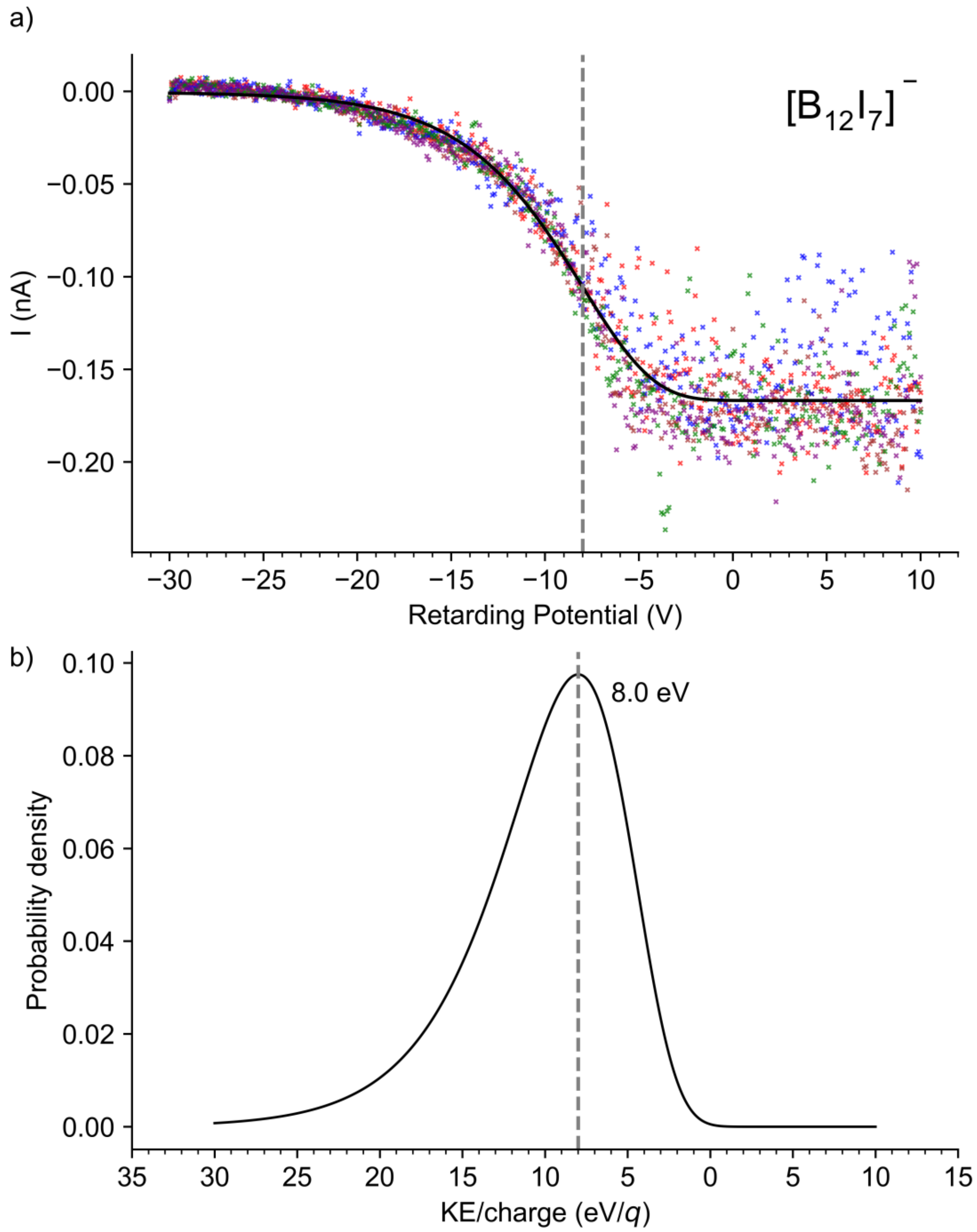

Figure S20. (a) Ion current of $[B_{12}I_7]^-$ as a function of the retarding potential. Five separate experiments under identical conditions were performed (individual data points are shown as "X" in a color representative for a single experiment) and all data was subsequently averaged and fitted by a sigmoidal function (black line). The position of the inflection point is marked with a dashed grey line. (b) Probability density function for the KE per charge (q) obtained by calculating the first derivative of the sigmoidal function in (a). The position of the maximum is marked with a dashed grey line and can be understood as most probable KE.

REFERENCES

(1) Farràs, P.; Vankova, N.; Zeonjuk, L. L.; Warneke, J.; Dülcks, T.; Heine, T.; Viñas, C.; Teixidor, F.; Gabel, D., From an Icosahedron to a Plane: Flattening Dodecaiodo-dodecaborate by Successive Stripping of Iodine. *Chem. Eur. J.* 2012, *18*, 13208-13212.
(2) Samayoa-Oviedo, H. Y.; Behrend, K.-A.; Kawa, S.; Knorke, H.; Su, P.; Belov, M. E.; Anderson, G.; Warneke, J.; Laskin, J., Design and Performance of a Soft-Landing Instrument for Fragment Ion Deposition. *Anal. Chem.* 2021, *93*, 14489-14496.
(3) Kawa, S.; Behrend, K. A.; Knorke, H.; Rohdenburg, M.; Volke, D.; Rothemund, S.; Warneke, J., Selective Functionalization of Peptides with Reactive Fragment Ions. *J. Am. Soc. Mass Spectrom.* 2025, *36*, 1779-1790.
(4) Su, P.; Hu, H.; Warneke, J.; Belov, M. E.; Anderson, G. A.; Laskin, J., Design and Performance of a Dual-Polarity Instrument for Ion Soft Landing. *Anal. Chem.* 2019, *91*, 5904-5912.
(5) Rohdenburg, M.; Kawa, S.; Ha-Shan, M.; Reichelt, M.; Knorke, H.; Denecke, R.; Warneke, J., Probing fragment ion reactivity towards functional groups on coordination polymer surfaces. *Chem. Commun.* 2024, *60*, 10306-10309.
